# The aryl hydrocarbon receptor contributes to tissue adaptation of intestinal eosinophils in mice

**DOI:** 10.1084/jem.20210970

**Published:** 2022-03-03

**Authors:** Nicola Laura Diny, Barbora Schonfeldova, Michael Shapiro, Matthew L. Winder, Sunita Varsani-Brown, Brigitta Stockinger

**Affiliations:** 1 The Francis Crick Institute, London, UK

## Abstract

Eosinophils are potent sources of inflammatory and toxic mediators, yet they reside in large numbers in the healthy intestine without causing tissue damage. We show here that intestinal eosinophils were specifically adapted to their environment and underwent substantial transcriptomic changes. Intestinal eosinophils upregulated genes relating to the immune response, cell–cell communication, extracellular matrix remodeling, and the aryl hydrocarbon receptor (AHR), a ligand-activated transcription factor with broad functions in intestinal homeostasis. Eosinophils from AHR-deficient mice failed to fully express the intestinal gene expression program, including extracellular matrix organization and cell junction pathways. AHR-deficient eosinophils were functionally impaired in the adhesion to and degradation of extracellular matrix, were more prone to degranulation, and had an extended life span. Lack of AHR in eosinophils had wider effects on the intestinal immune system, affecting the T cell compartment in nave and helminth-infected mice. Our study demonstrates that the response to environmental triggers via AHR partially shapes tissue adaptation of eosinophils in the small intestine.

## Teaser

Eosinophils undergo tissue adaptation in the small intestine. Aryl hydrocarbon receptor–deficient eosinophils fail to induce the full intestinal gene expression program, are functionally impaired in extracellular matrix interactions, are more prone to degranulation, and have an extended life span.

## Graphical Abstract

**Figure:**
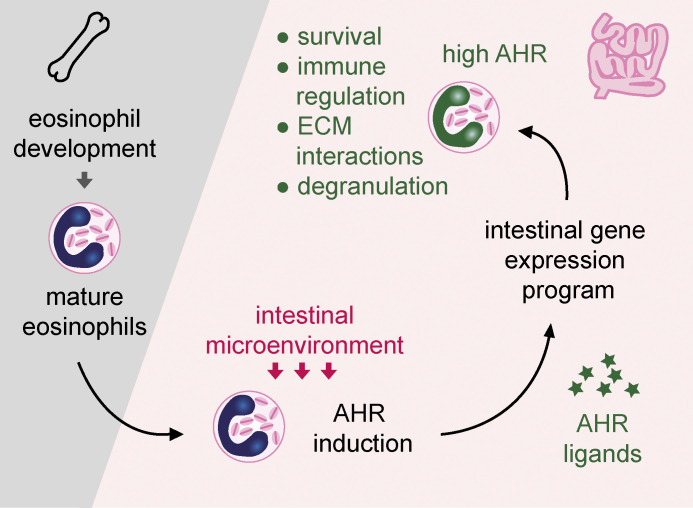

## Introduction

Intestinal immune cells are specially adapted to tolerate exposure to the commensal microbiota while maintaining the ability to mount a potent immune response to pathogens. This process of tissue adaptation has long been studied in lymphoid cells and more recently in macrophages (Faria et al., 2017; Mowat et al., 2017). Eosinophils are one of the most numerous immune cells in the small intestine (Powell et al., 2010). Although they are more versatile than the end-stage effector cells as which they are classically portrayed, they nevertheless have the potential to cause inflammation and tissue damage (Lee et al., 2010; Rothenberg and Hogan, 2006). This suggests that the intestinal microenvironment prevents them from doing so and begs the question whether eosinophils undergo tissue adaptation.

Following development in the bone marrow, mature eosinophils are released into the bloodstream from where they migrate into tissues such as the thymus, uterus, and small intestine (Mishra et al., 1999). Eosinophils are abundant in the small intestine and to a lesser extent in the colon of mice and humans under homeostatic conditions (Chojnacki et al., 2019; DeBrosse et al., 2006; Matsushita et al., 2015; Sugawara et al., 2016). Their lifetime in tissues is variable, with a half-life of <1 d in blood and lung and ∼6 d in the small intestine (Carlens et al., 2009). Parasitic infections and allergic or chronic inflammatory diseases lead to increased eosinophil infiltration, and eosinophils contribute to disease pathology (Busse and Sedgwick, 1992; Filippone et al., 2018; Furuta et al., 2005; Mehta and Furuta, 2015; Smyth et al., 2013; Yantiss, 2015). Nevertheless, eosinophils may also regulate inflammation (Masterson et al., 2015), and their functional potential is wide-ranging. Eosinophils have antiviral (Drake et al., 2016; Rosenberg and Domachowske, 2001; Sabogal Pineros et al., 2019), antibacterial (Arnold et al., 2018; Krishack et al., 2019; Linch et al., 2009), and antiparasitic properties (Huang et al., 2015; Specht et al., 2006; Turner et al., 2018) and modulate immune responses (Rosenberg et al., 2013). They contribute to tissue remodeling and repair as well as fibrosis (Coden and Berdnikovs, 2020; Lee et al., 2004; Minshall et al., 1997; Pegorier et al., 2006; Takemura et al., 2018) and can promote coagulation (Uderhardt et al., 2017).

Despite this range of known eosinophil functions, their physiological role in the intestine is not well understood. Studies are emerging that suggest eosinophils may affect the intestinal barrier (Buonomo et al., 2016; Johnson et al., 2015; Singh et al., 2019), but definitive mechanisms are lacking to date. Eosinophils can suppress T helper 1 (Th1) and Th17 cells in the small intestine by expressing PD-L1 and by secreting IL-1 receptor antagonist (Arnold et al., 2018; Sugawara et al., 2016) or promote T regulatory cells (Chen et al., 2015). Initial reports that eosinophils promote IgA production and plasma cell maintenance (Chu et al., 2014b; Chu et al., 2011; Jung et al., 2015) have since been refuted and explained by the use of mice from different vendors rather than littermate controls (Beller et al., 2020; Bortnick et al., 2018; FitzPatrick et al., 2020; Haberland et al., 2018; Singh et al., 2019). This highlights the need for careful characterization of eosinophils in the steady-state intestine.

The aryl hydrocarbon receptor (AHR) is a ligand-activated transcription factor that regulates transcriptional programs in response to environmental ligands (Stockinger et al., 2021; Yang et al., 2018). It interacts with several other pathways including NF-κB, retinoic acid receptor, and Wingless and Int-1 (WNT) signaling pathways (Mathew et al., 2008; Murphy et al., 2007; Park et al., 2018). The intestine is constantly exposed to AHR ligands derived from the diet and microbial metabolism (Schanz et al., 2020), and AHR plays key roles in the epithelial and immune compartments to maintain barrier function and intestinal homeostasis (Lamas et al., 2018; Li et al., 2011; Metidji et al., 2018; Qiu et al., 2012; Schiering et al., 2017; Stockinger et al., 2014). In myeloid cells, AHR suppresses inflammatory cytokine production in LPS shock models (Kimura et al., 2009; Nguyen et al., 2010; Thatcher et al., 2016) and favors differentiation of monocytes into dendritic cells over macrophages (Goudot et al., 2017). Its role in eosinophils, however, has not been studied. Here, we identify AHR as a key regulator of eosinophil survival, granularity, and extracellular matrix (ECM) interactions in the small intestine. We find that eosinophils undergo large transcriptional changes from the bone marrow to the small intestine, and this tissue adaptation is in part controlled by AHR.

## Results

### Eosinophils undergo tissue adaptation in the small intestine

**Figure 1.**
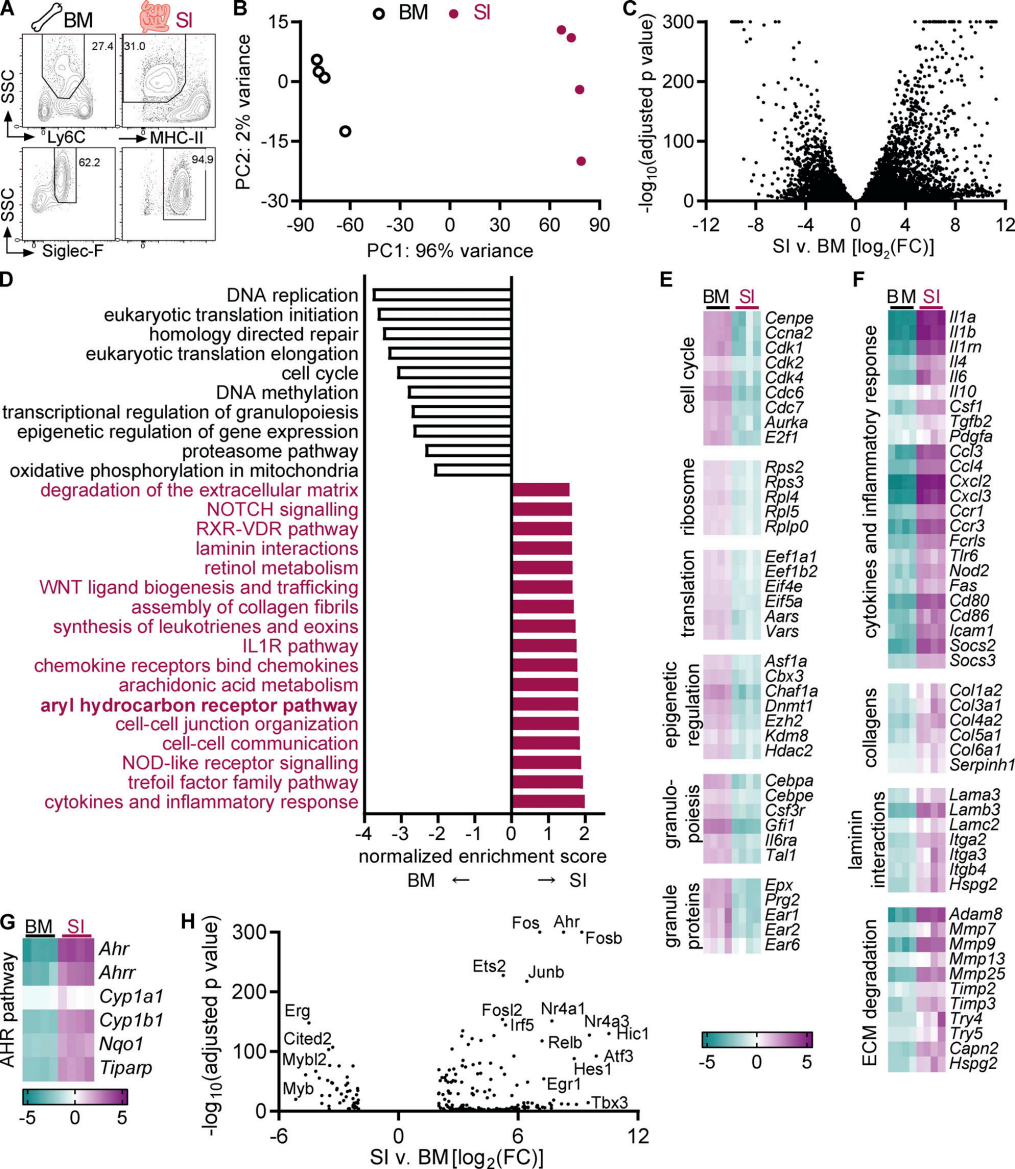
**Eosinophils undergo tissue adaptation in the small intestine.** RNA sequencing was conducted on sorted eosinophils from the bone marrow and small intestine of *n* = 4 female mice each. **(A)** Eosinophils were sorted from the bone marrow and small intestine. Full gating strategy is depicted in Fig. S1, A–C. **(B)** Principal component analysis of RNA sequencing data. **(C)** Volcano plot of differentially expressed genes. For adjusted P values = 0, the value of 10^–300^ was assigned. **(D)** Canonical pathways enriched in eosinophils from the small intestine compared with bone marrow eosinophils were determined by GSEA. A selection of pathways with the highest enrichment scores are shown. **(E–G)** Examples of functionally defined gene subsets that are differentially expressed between small intestinal and bone marrow eosinophils (adjusted P value <0.05). Shown is the log_2_ fold change to the geometric mean of TPM + 1. **(H)** Volcano plot of differentially expressed transcription factors (|FC| > 4, adjusted P < 0.05). BM, bone marrow; SI, small intestine; TPM, transcripts per million.

**Figure S1.**
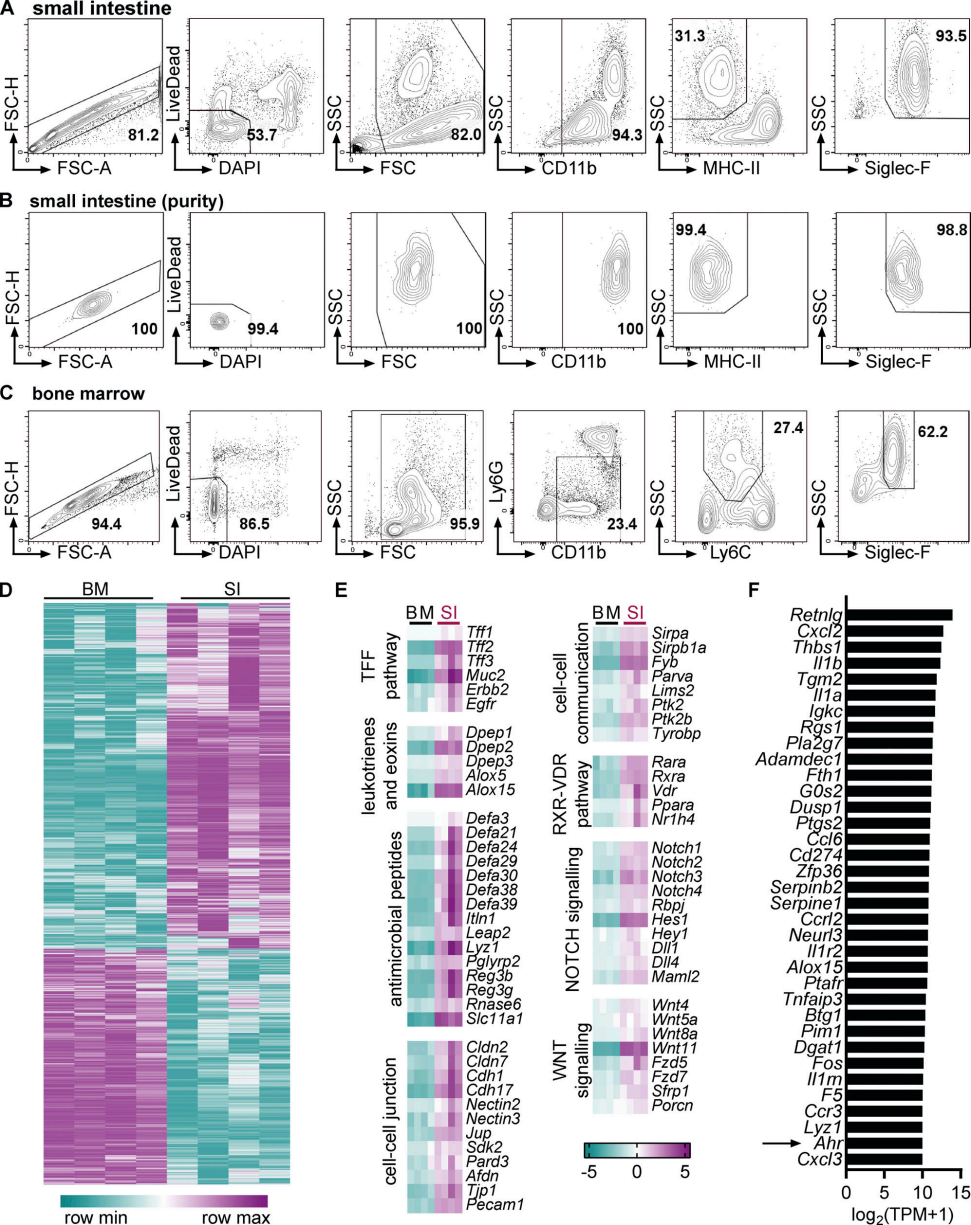
**Comparison of the eosinophil transcriptome between bone marrow and small intestine. (A–C)** Representative flow cytometry plots depicting gating strategy for eosinophil sorting from SI (A and B) and bone marrow (C) for RNA sequencing. Intestinal cells were positively enriched with anti-CD11b microbeads before FACS sorting. **(B)** Representative flow cytometry plots depicting eosinophil purity after sorting. **(D)** Heatmap of differentially expressed genes. **(E)** Examples of functional groups of genes with positive enrichment in small intestinal eosinophils. Log_2_(FC) to geometric mean of TPM + 1 is shown. **(F)** Genes with highest absolute expression (TPM) in small intestinal eosinophils that were also differentially expressed compared with bone marrow eosinophils with a FC >10. BM, bone marrow; SI, small intestine; TPM, transcripts per million.

To gain a better understanding of intestinal eosinophils, we compared their transcriptome to that of bone marrow eosinophils. Eosinophils were FACS-sorted from the intestine (CD11b^+^MHC-II^–^SiglecF^+^SSC^hi^) and bone marrow (CD11b^+^Ly6G^–^Ly6C^–^SiglecF^+^SSC^hi^) after gating of live, single cells (Fig. 1 A and Fig. S1, A–C). Principal component analysis of the RNA sequencing data revealed extensive changes in the eosinophil transcriptome between bone marrow and small intestine (Fig. 1 B). Eosinophils clustered according to tissue, which accounted for 96% of variance within the dataset. >13,000 genes were differentially expressed, and >450 genes were upregulated >100-fold in small intestinal eosinophils (Figs. 1 C and S1 D).

Gene set enrichment analysis (GSEA) identified canonical pathways that were enriched in the two eosinophil populations (Fig. 1 D). Bone marrow eosinophils were enriched for genes involved in DNA replication and repair, supporting the concept that intestinal eosinophils are postmitotic cells (Fig. 1 E). Multiple enriched pathways in bone marrow eosinophils related to protein translation. During development, eosinophils preform large quantities of granule proteins, cytokines, and more for subsequent release in tissues (Weller and Spencer, 2017). This extensive protein production also leads to an induction of the unfolded protein response (Bettigole et al., 2015) and proteasomal degradation pathways. Pathways concerning transcriptional and epigenetic regulation of gene expression were enriched in bone marrow eosinophils, suggesting active remodeling of the chromatin landscape. Enrichment of the oxidative phosphorylation pathway points to changes in the metabolism between bone marrow and intestinal eosinophils. As previously reported, eosinophils from the bone marrow expressed higher levels of the main granule proteins and eosinophil-associated ribonucleases (Fig. 1 E; Attery and Batra, 2017; Cormier et al., 2001).

Several pathways with positive enrichment in intestinal eosinophils were related to the immune response, such as cytokines and inflammatory mediators, chemokine signaling, or nucleotide-binding and oligomerization domain (NOD)–like receptor signaling (Fig. 1, D and F). Small intestinal eosinophils also showed increased expression of microbial recognition and defense pathways, including the trefoil factor family (Hoffmann, 2004; Mashimo et al., 1996) and many antimicrobial peptides (Fig. S1 E). The IL1R pathway was enriched and, in agreement with a previous study (Sugawara et al., 2016), *Il1a*, *Il1b*, and *Il1rn* (IL1R antagonist) were among the most highly expressed genes in intestinal eosinophils (Figs. 1 F and S1 F). Genes functioning in cell–cell communication and junction formation were increased in intestinal eosinophils (Figs. 1 D and S1 E), demonstrating that eosinophils interact with other cells in the lamina propria. Intestinal eosinophils had increased expression of genes related to fibrosis, including ECM components such as collagens and laminins, but also proteins functioning in ECM remodeling and degradation (Fig. 1, D and F). It was previously reported that eosinophils contribute to fibrosis in the intestine after injury (Takemura et al., 2018). The enrichment of these pathways indicates that eosinophils actively remodel the ECM during homeostasis as well. Taken together, these findings show that eosinophils actively shape the small intestinal microenvironment through cytokines and lipid mediators, direct cell–cell contact, and modification of the ECM.

Another highly enriched gene set in intestinal eosinophils was the AHR pathway (Fig. 1 D). The *Ahr* gene itself and AHR canonical target genes Ahr repressor (*Ahrr*), cytochrome P450 enzymes *Cyp1b1* and *Cyp1a1*, NAD(P)H dehydrogenase (quinone) 1 (*Nqo1*), and tetrachlorodibenzo-p-dioxin-inducible poly(ADP-ribose) polymerase (*Tiparp*) were all increased in small intestinal eosinophils in the RNA-sequencing dataset (Fig. 1 G). *Ahr* was also among the most upregulated transcription factors in small intestinal eosinophils (Fig. 1 H) and among the differentially expressed genes with highest absolute expression (Fig. S1 F). This is of particular interest given the important role AHR plays in the physiological function of other intestinal immune cells, its ability to affect gene expression on a large scale, and its known interactions with other pathways that were enriched in intestinal eosinophils such as the WNT and retinoic acid signaling pathways (Figs. 1 D and S1 E). This prompted us to further analyze the AHR pathway in intestinal eosinophils and led us to hypothesize that AHR may affect the function and tissue adaptation of eosinophils in the small intestine.

### AHR is induced in intestinal eosinophils

**Figure S2.**
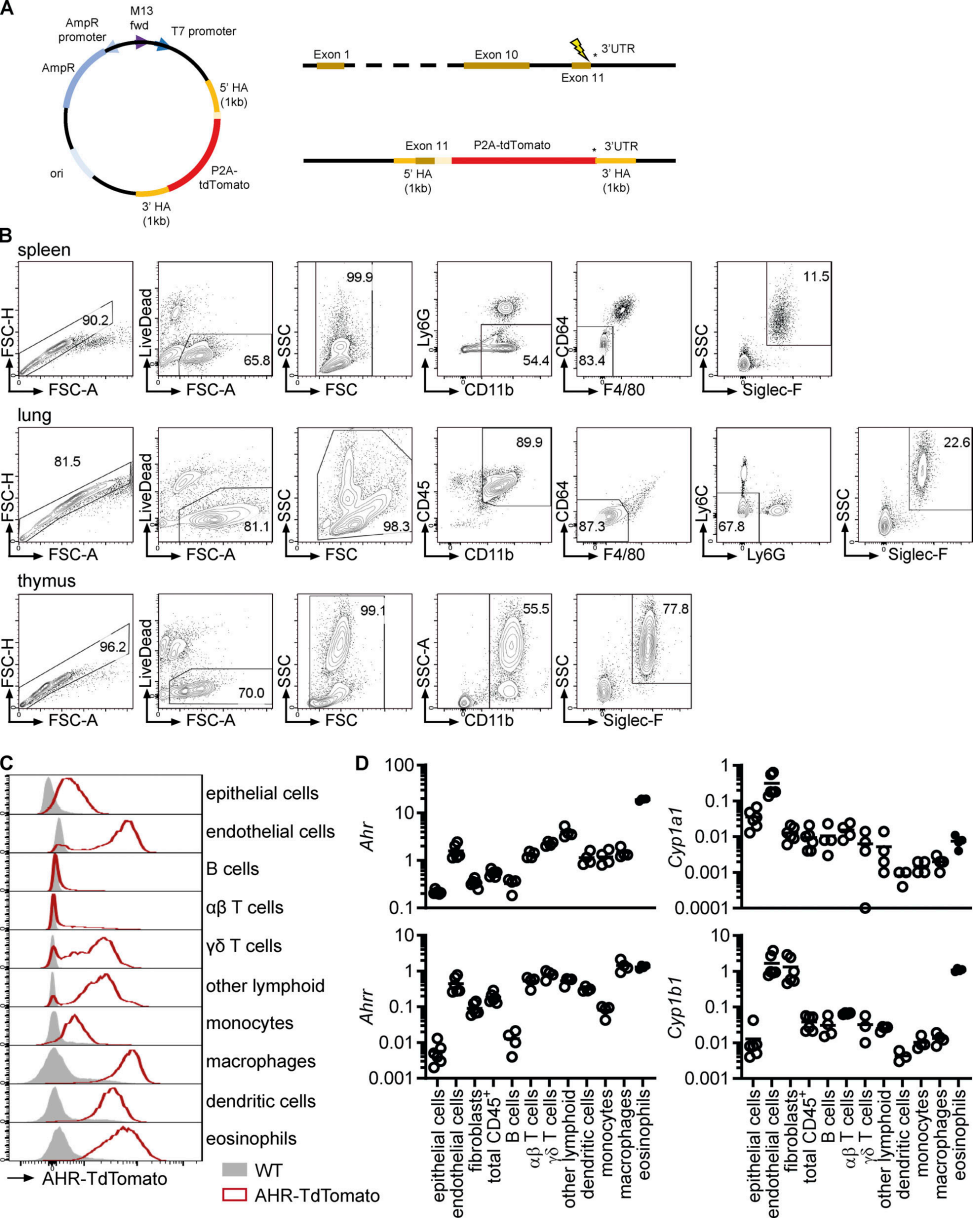
**AHR expression in intestinal eosinophils. (A)** Schematic of generation of AHR-TdTomato mice including DNA donor repair template, Ahr locus, and Ahr-P2A-tdTomato targeted locus. **(B)** Eosinophil gating and sorting strategy from different tissues for qPCR and flow cytometric analysis. Cells were positively enriched with anti-CD11b microbeads before sorting. Eosinophils from bone marrow and small intestine were sorted as shown in Fig. S1, A–C, using only LiveDead stain for dead cell exclusion. **(C)** AHR-TdTomato expression across different small intestinal cell types was determined by flow cytometry. Histograms are representative of *n* = 4 AHR-TdTomato mice from two independent experiments. **(D)** Gene expression was determined by qPCR across different cell types sorted from the small intestine of WT mice. Gene expression was normalized to *Hprt*. Data are from one experiment with four to six biological replicates. Missing data points indicate that the gene was not detectable by qPCR.

**Figure 2.**
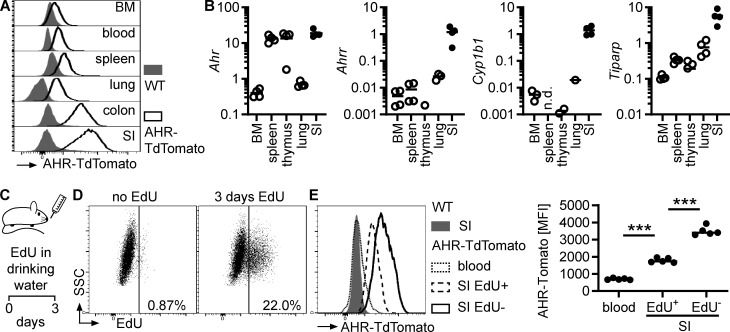
**AHR is induced in intestinal eosinophils. (A)** AHR-TdTomato expression in eosinophils from different tissues was determined by flow cytometry. Histograms are representative of *n* = 4 AHR-TdTomato mice from two independent experiments. **(B)** Gene expression in eosinophils FACS-sorted from different tissues was determined by qPCR and normalized to *Hprt*. Data are representative of two independent experiments with *n* = 3–4 mice per tissue. Missing data points indicate RNA was not detectable by qPCR. **(C–E)** AHR-TdTomato mice were treated with EdU in drinking water for 3 d. AHR-TdTomato fluorescence in eosinophils from the blood and small intestine was quantified by flow cytometry. Data are representative of two independent experiments with *n* = 3–5 mice. One-way ANOVA; ***, P < 0.001. BM, bone marrow; MFI, mean fluorescence intensity; n.d., not detected; SI, small intestine.

We generated AHR reporter mice (AHR-TdTomato; Fig. S2 A) to analyze AHR expression in eosinophils from different tissues. AHR-TdTomato expression in eosinophils was lowest in the bone marrow and blood, increased in other tissues, and reached the highest expression in small intestinal eosinophils (Fig. 2 A). This was confirmed by quantitative PCR (qPCR) in eosinophils sorted from different tissues (Figs. S2 B and 2 B). Furthermore, small intestinal eosinophils had particularly high AHR target gene expression (*Ahrr*, *Cyp1b1*, and *Tiparp*) compared with eosinophils from any other tissue (Fig. 2 B). This was likely a combination of both high AHR expression and the abundance of AHR ligands in the small intestine. AHR-TdTomato reporter activity was detectable in all small intestinal cell types analyzed, although the fluorescence intensity varied substantially between cell types (Fig. S2 C). Eosinophils, but also macrophages, dendritic cells, and endothelial cells, showed particularly high AHR expression. We confirmed this finding by FACS-sorting different intestinal populations and analyzing expression of *Ahr* and AHR target genes by qPCR (Fig. S2 D). To determine when eosinophils upregulate AHR, we treated AHR-TdTomato mice with 5-ethynyl-2′-deoxyuridine (EdU) for 3 d (Fig. 2 C). Since eosinophils are not thought to proliferate in the small intestine under steady-state conditions, this should label newly migrated small intestinal eosinophils. After 3 d, ∼20% of small intestinal eosinophils were EdU^+^ (Fig. 2 D). Recently migrated EdU^+^ eosinophils had higher AHR expression than blood eosinophils but lower AHR expression than older EdU^–^ small intestinal eosinophils (Fig. 2 E). This suggests that the intestinal microenvironment causes AHR induction in eosinophils.

### Eosinophils activate the canonical AHR pathway in response to AHR ligands

**Figure 3.**
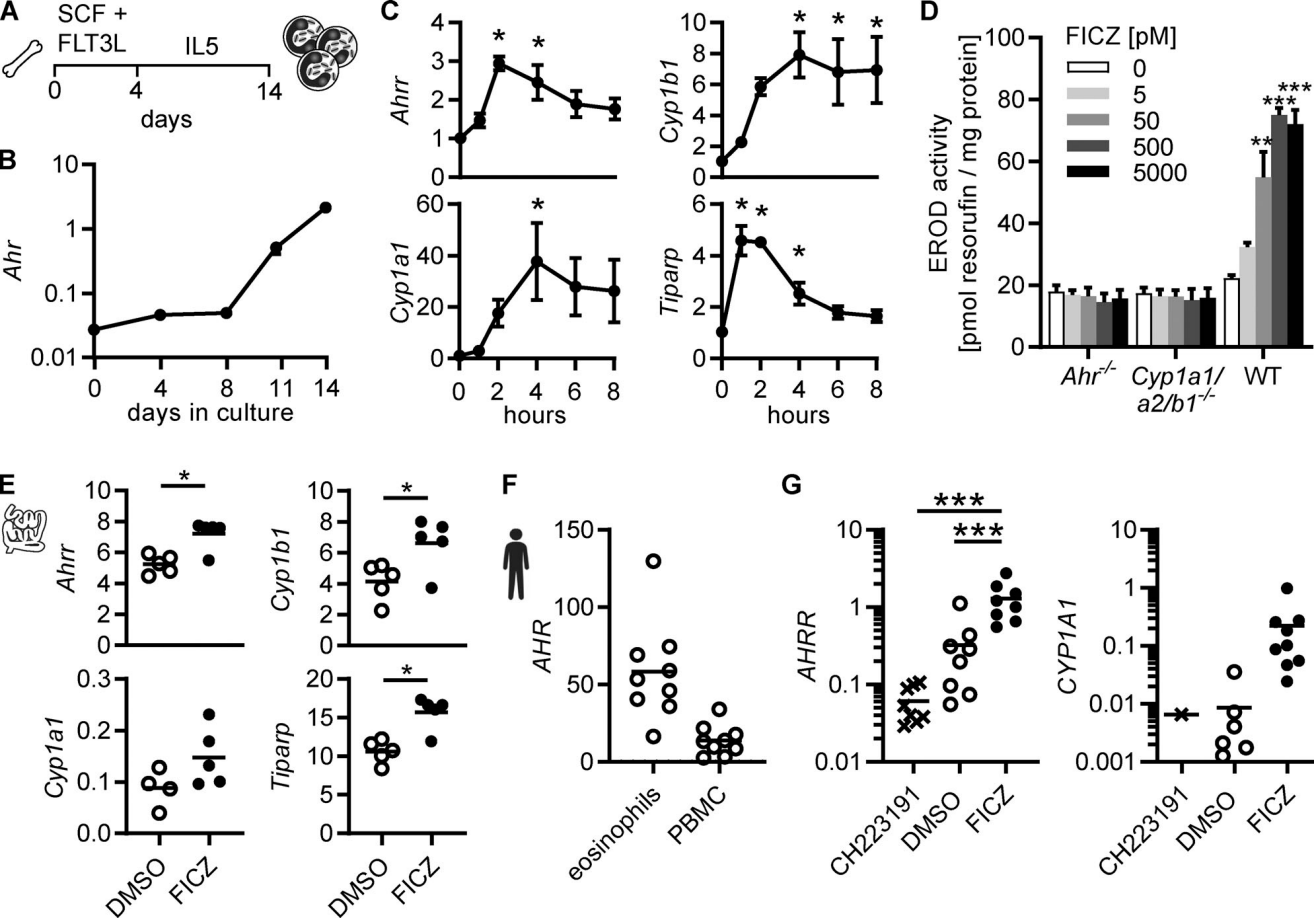
**Eosinophils activate the canonical AHR pathway in response to AHR ligands. (A)** Schematic of BMDEo cultures. **(B)** Gene expression in BMDEo at different timepoints in culture was determined by qPCR and normalized to *Hprt*. Shown is the mean ± SEM of three independent experiments. **(C)** BMDEo were cultured to day 14 and treated with 5 nM FICZ or DMSO for indicated times. qPCR of AHR target genes was normalized to *Hprt* and DMSO-treated controls. Data are mean ± SEM of *n* = 4 independent experiments. *, P < 0.05, one-way ANOVA to DMSO control. **(D)** BMDEo of different genotypes were cultured to day 14 and treated with different concentrations of FICZ or DMSO control for 4 h before ethoxyresorufin-*O*-deethylase activity was measured. Data are mean ± SEM of *n* = three independent experiments. **, P < 0.01; ***, P < 0.001, one-way ANOVA to DMSO control. **(E)** Small intestinal eosinophils were FACS-sorted and cultured in vitro in the presence of 5 nM FICZ or DMSO control and 2 ng/ml IL-5 for 2 h. Gene expression was determined by qPCR and normalized to *Hprt*. *, P < 0.05, paired *t* test. Data are representative of two independent experiments with *n* = 4–5 mice. **(F and G)** Human eosinophils and PBMCs were isolated from blood of normal donors. Gene expression was analyzed by qPCR and normalized to *HPRT*. **(F)** Eosinophils and PBMCs were cultured for 4 h with DMSO. **(G)** Eosinophils were cultured with DMSO, 3 μm CH223191, or 5 nM FICZ for 4 h. *CYP1A1* expression was detectable in only one of nine CH223191-treated and in six of nine DMSO-treated samples. Data are from nine donors. ***, P < 0.001, repeated-measure one-way ANOVA.

Eosinophils can be generated in vitro by culturing bone marrow cells with stem cell factor and FLT3L for 4 d, followed by differentiation into eosinophils with IL-5 for another 10 d (Fig. 3 A). In these bone marrow–derived eosinophil (BMDEo) cultures, *Ahr* expression increased over time (Fig. 3 B). Treatment of mature BMDEo on day 14 of culture with the AHR ligand 6-formylindolo[3,2-*b*]carbazole (FICZ) resulted in a further, transient upregulation of canonical AHR target genes (Fig. 3 C) and a dose-dependent increase in CYP1A1 enzymatic activity (Fig. 3 D). In small intestinal eosinophils, expression of AHR target genes could be further induced by ex vivo culture with FICZ (Fig. 3 E). *AHR* was also expressed in human eosinophils. qPCR analysis of human eosinophils isolated from the blood of healthy donors readily detected *AHR* (Fig. 3 F), and human eosinophils induced *AHRR* and *CYP1A1* after 4-h culture with FICZ (Fig. 3 G). The AHR inhibitor CH223191 suppressed *AHRR* and *CYP1A1* expression. This demonstrates that eosinophils from mice and humans express functional AHR and respond to AHR ligands with the induction of canonical target genes.

### AHR deficiency alters the transcriptome of intestinal eosinophils

**Figure 4.**
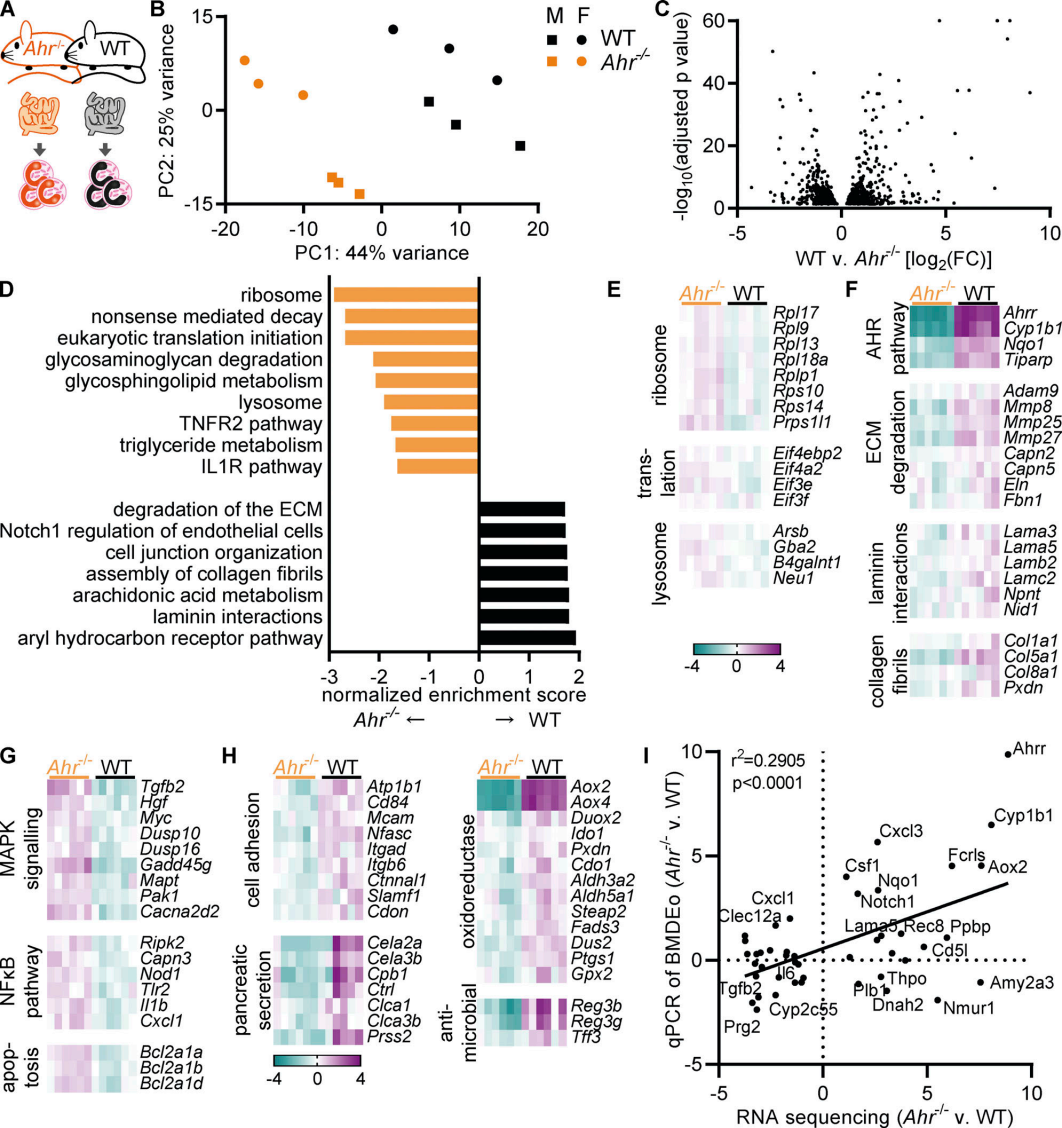
**AHR deficiency alters the transcriptome of intestinal eosinophils. (A)** RNA sequencing of small intestinal eosinophils from WT and *Ahr*^–/–^ mice was performed in *n* = 3 males and *n* = 3 females per genotype. **(B)** Principal component analysis. **(C)** Volcano plot of differentially expressed genes between WT and *Ahr*^–/–^ eosinophils. **(D)** GSEA of WT versus *Ahr*^–/–^ eosinophils. **(E and F)** Examples of functionally defined gene subsets from the leading edge of the analysis in D. **(G and H)** Examples of functionally defined gene subsets identified by DAVID (Fig. S3, E and F) based on differentially expressed genes between WT and *Ahr*^–/–^ eosinophils (|FC| > 2, adjusted *P* value <0.05). **(E–H)** Shown is the log_2_(FC) to the geometric mean of TPM + 1. **(I)** Genes with differential expression between WT and *Ahr*^–/–^ were manually selected and analyzed by qPCR in BMDEo. Correlation of RNA sequencing results with qPCR results for WT versus *Ahr*^–/–^ BMDEo. Shown is log_2_(FC). qPCR data are from one experiment conducted in technical triplicates.

**Figure S3.**
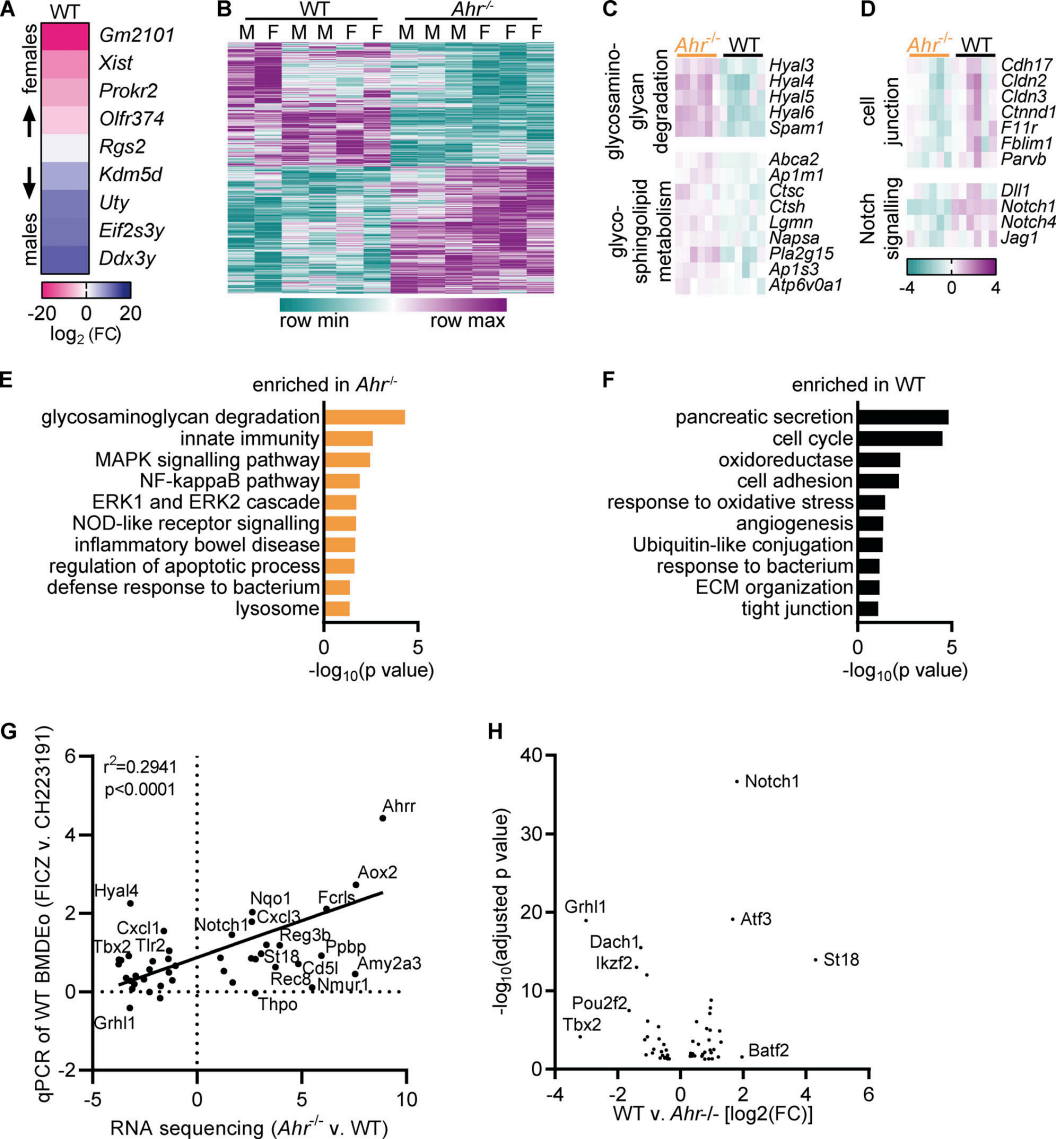
**AHR deficiency alters the transcriptome of intestinal eosinophils. (A)** Differentially expressed genes between eosinophils from the small intestine of male and female WT mice with an adjusted P value <0.1. **(B)** Heatmap of differentially expressed genes (adjusted P value <0.05) between WT and *Ahr*^–/–^ eosinophils from the small intestine. **(C and D)** Examples of functionally defined gene subsets from the leading edge of the analysis in Fig. 4 D. Shown is the log_2_(FC) to the geometric mean of TPM + 1. **(E and F)** Enriched pathways (P < 0.1) identified by DAVID based on differentially expressed genes between WT and *Ahr*^–/–^ eosinophils (|FC| > 2, adjusted *P* value <0.05). **(G)** From the RNA sequencing dataset of small intestinal eosinophils, 55 genes with differential expression between WT and *Ahr*^–/–^ were manually selected and analyzed by qPCR in BMDEo. Of those, 11 genes were undetectable in BMDEo. Correlation of RNA sequencing results with qPCR results is shown for WT BMDEo treated for 4 h with 5 nM FICZ versus 3 μM CH223191. Shown is log_2_(FC). qPCR data are from one experiment conducted in technical triplicate. **(H)** Volcano plot of transcription factors with differential expression between WT and *Ahr*^–/–^ eosinophils.

Beyond the canonical pathway, AHR can affect the expression of thousands of genes (Yang et al., 2018). To gain further insight into the function of AHR in eosinophils, we analyzed the transcriptome of small intestinal eosinophils from *Ahr*^–/–^ and WT mice (Fig. 4 A). Samples from male and female mice were included to account for possible sex differences, but only a few genes were differentially expressed between male and female samples (Fig. S3 A). Differences between genotypes were much stronger. Principal component analysis showed that *Ahr*^–/–^ eosinophils clustered away from WT mice (Fig. 4 B). Accordingly, 1,292 genes were differentially expressed between WT and *Ahr*^–/–^ eosinophils (Figs. 4 C and S3 B). Of those, 281 genes were upregulated and 176 genes downregulated more than twofold. GSEA revealed the effect of AHR on multiple biological pathways (Fig. 4 D), many of which were also enriched in the comparison of eosinophils from bone marrow and small intestine (Fig. 1 D). Intestinal eosinophils from *Ahr*^–/–^ mice showed similarity with bone marrow eosinophils, as they were enriched for ribosomal genes and those involved in translation (Fig. 4, D and E), although the differences in gene expression levels were small (<1.5-fold). *Ahr*^–/–^ eosinophils also showed increased expression of lysosomal genes (Fig. 4 E) and genes relating to the metabolism of glycosaminoglycans, glycosphingolipids, and triglycerides (Fig. S3 C). The IL1R and TNFR2 pathways were enriched as well, suggesting a possible increase in proinflammatory signaling.

In WT eosinophils, enriched pathways overlapped with those enriched in small intestinal eosinophils (Figs. 4 D and 1 D). As expected, the AHR canonical pathway showed strong differential expression (Fig. 4, D and F). WT eosinophils had increased expression of genes functioning in ECM degradation, as well as ECM components such as collagen fibrils and laminins, indicating that AHR-deficient eosinophils could be less competent in remodeling the ECM. WT eosinophils were also enriched in cell junction–related genes and the Notch pathway (Fig. S3 D). All of these pathways were also upregulated in the comparison of small intestinal to bone marrow eosinophils. This strongly points to AHR being required for the full expression of intestine-adapted genes in eosinophils.

Many of the pathways identified by GSEA included genes with small fold changes (FCs). To identify the function of the most differentially expressed genes between WT and *Ahr*^–/–^ eosinophils, the online database for annotation, visualization, and integrated discovery (DAVID) was used to annotate genes with |FC| > 2. This revealed additional pathways in both genotypes (Fig. S3, E and F). *Ahr*^–/–^ eosinophils showed increased expression of NOD-like receptor and inflammatory pathways (Figs. 4 G and S3 E). They also upregulated genes that may result in increased survival. The MAPK pathway is active in surviving eosinophils (Kankaanranta et al., 1999) and increased in *Ahr*^–/–^ eosinophils. Increased activation of the NF-κB pathway as well as increased expression of inflammatory mediators such as *Il1b* and *Cxcl1*, and of the antiapoptotic Bcl2-related protein A1 genes (Fig. 4 G), suggest these cells are hyperresponsive to signals stimulating the NF-κB pathway. In WT eosinophils, additional pathways relating to cell adhesion and tight junctions were identified (Figs. 4 H and S3 F). WT eosinophils had strongly increased expression of numerous proteases (identified as “pancreatic secretion”) and of a group of oxidoreductase enzymes. Antimicrobial peptides important for the intestinal barrier such as *Reg3b*, *Reg3g*, and *Tff3* were also increased in WT eosinophils. These findings advocate for AHR as a regulator of eosinophils’ ability to interact with other cells and to produce antimicrobial peptides.

To determine whether the observed transcriptional changes in AHR-deficient eosinophils were cell intrinsic, 55 genes with differential expression in small intestinal eosinophils were selected and analyzed in BMDEo cultures from WT and *Ahr*^–/–^ mice. Of those, 11 were not detectable in BMDEo, suggesting that the intestinal microenvironment is necessary for their expression. For the remaining genes, good correlation of the AHR dependence between BMDEo and small intestinal eosinophils was observed (Fig. 4 I), implying that many of the transcriptional changes observed in small intestinal eosinophils from WT and *Ahr*^–/–^ mice are likely cell intrinsic. Next, the ability of the AHR ligand FICZ to induce these genes was tested. Most of the genes that were downregulated in *Ahr*^–/–^ small intestinal eosinophils could be induced by FICZ stimulation in WT BMDEo (Fig. S3 G). This shows that ligand stimulation in eosinophils can induce AHR-dependent genes beyond the canonical pathway. In contrast, FICZ did not suppress genes upregulated in *Ahr*^–/–^. AHR may affect their gene expression indirectly by changing the expression of other transcription factors (Fig. S3 H). Taken together, these findings demonstrate that AHR regulates the expression of hundreds of genes and associated functional pathways in intestinal eosinophils.

### Intestinal tissue adaptation of eosinophils is partially controlled by AHR

**Figure 5.**
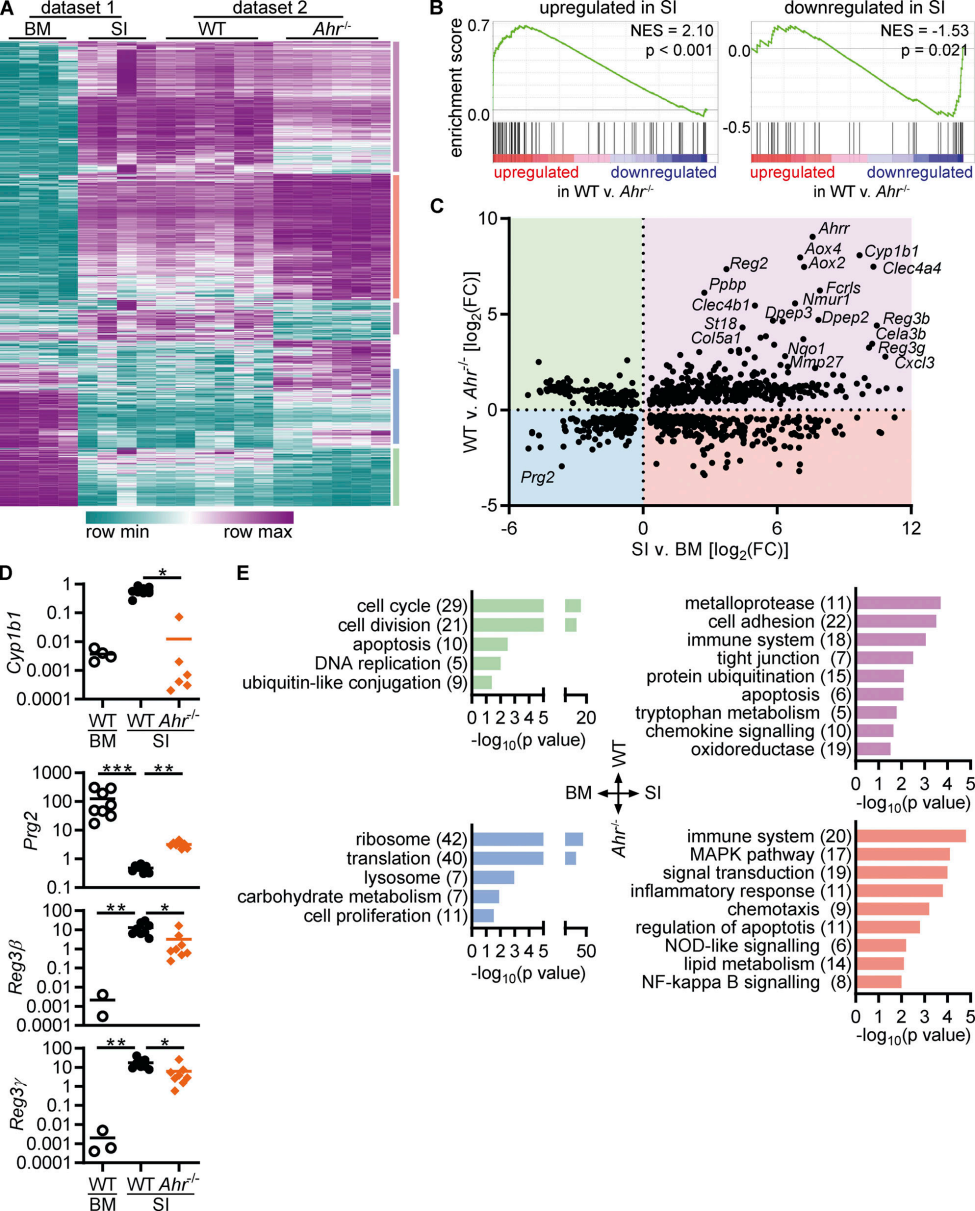
**Intestinal tissue adaptation of eosinophils is partially controlled by AHR. (A)** Hierarchical clustering of genes with differential expression in WT versus *Ahr*^–/–^ eosinophils after normalization of dataset 1 to dataset 2. **(B)** Enrichment of the 100 most up- or downregulated genes from the comparison of intestinal versus bone marrow eosinophils in the dataset of WT versus *Ahr*^–/–^ intestinal eosinophils. **(C)** Correlation of gene expression between the two RNA sequencing datasets. Only genes with differential expression in both datasets are shown. **(D)** Expression of selected genes was confirmed by qPCR in sorted eosinophils from BM and SI of different mice. Data are normalized to *Hprt* and are from *n* = 8 mice per group. Missing data points indicate that the gene was not detectable by qPCR. **(E)** Enriched pathways (P < 0.05) identified by DAVID based on differentially expressed genes (adjusted P value <0.05). Numbers in parentheses indicate the number of genes in each pathway. Subsets correspond to the four quadrants in C. *, P < 0.05; **, P < 0.01; ***, P, < 0.001; Kruskal-Wallis test with Dunn's correction for multiple comparison. BM, bone marrow; SI, small intestine.

The high expression of AHR in intestinal eosinophils and the overlap between biological pathways enriched in our two datasets support the idea that AHR is an important factor for intestinal tissue adaptation of eosinophils. To visualize changes across the two tissues and genotypes, the datasets were combined, and genes with differential expression were clustered. Most genes that were affected by the lack of AHR also changed from the bone marrow to the small intestine (1,046 of 1,292 genes) demonstrating that AHR-regulated genes are overwhelmingly part of the tissue adaptation program (Fig. 5 A). Using GSEA, we found that the 100 most upregulated genes in eosinophils from the small intestine versus bone marrow were enriched in WT eosinophils, whereas the 100 most downregulated genes were enriched in *Ahr*^–/–^ eosinophils (Fig. 5 B). This demonstrates that AHR-dependent changes to the eosinophil transcriptome are part of the intestinal tissue adaptation.

We correlated differentially expressed genes from the two RNA-sequencing datasets to determine how AHR affects the tissue adaptation program of intestinal eosinophils. Most genes with differential expression in both WT versus *Ahr*^–/–^ eosinophils and small intestinal versus bone marrow eosinophils were upregulated in the small intestine (720 of 1,046 genes; Fig. 5 C). We confirmed the expression of some of these genes by qPCR (Fig. 5 D). Antimicrobial peptides *Reg3b* and *Reg3g* and the classic AHR target *Cyp1b1* are upregulated in intestinal eosinophils and in WT compared with *Ahr*^–/–^ eosinophils. Conversely, the granule protein–encoding gene *Prg2* was among the genes that are downregulated in the intestine and in WT eosinophils. Using DAVID, the enrichment of biological pathways in genes of all four subsets (Fig. 5 C) was analyzed. The resulting pathways were very similar to those identified earlier by analyzing the datasets individually (Figs. 5 E, 1 D, and 4 D). Among genes upregulated in the bone marrow, pathways relating to cell cycle and the ubiquitin-proteasome system were enriched in WT eosinophils, while protein translation was enriched in *Ahr*^–/–^ eosinophils. Of particular interest were the genes with high expression in the small intestine and in WT eosinophils, as they are AHR dependent and tissue specific. This subset also showed the highest (>100) FCs (Fig. 5 C). Enriched pathways in this subset included those identified earlier, such as metalloproteases, cell adhesions and tight junctions, oxidoreductase enzymes, and tryptophan metabolism (Fig. 5 E). Immune system–related genes were generally upregulated in the small intestine, some of them increased in WT eosinophils and others were increased in *Ahr*^–/–^ eosinophils. Among those enriched in *Ahr*^–/–^ eosinophils in particular were inflammatory response genes and genes involved in NF-κB signaling. Taken together, these data demonstrate an important role for AHR in mediating part of the tissue adaptation of eosinophils to the small intestine.

### AHR controls eosinophil survival in the small intestine

**Figure 6.**
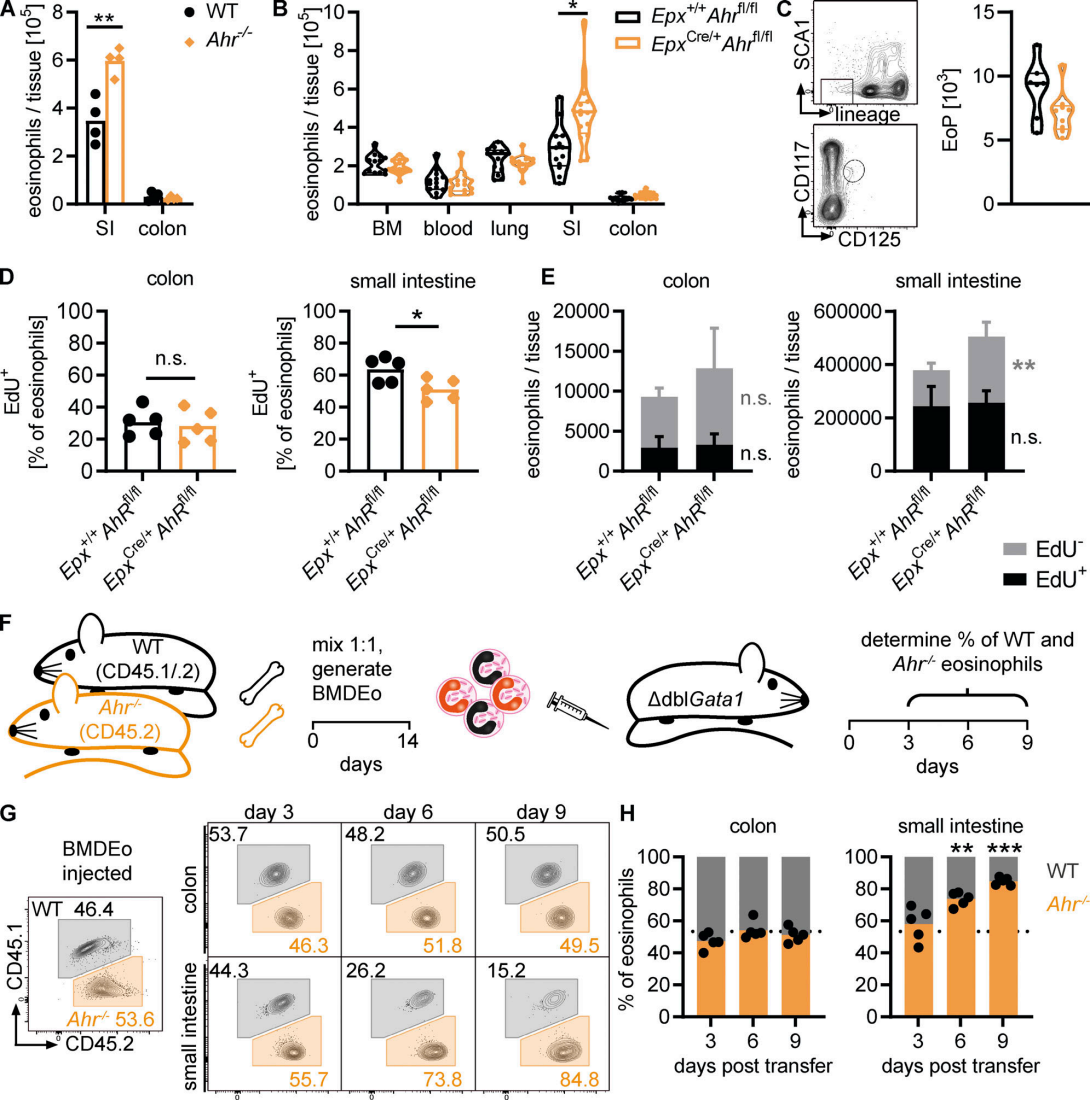
**AHR regulates eosinophil survival in the small intestine. (A)** Eosinophils were quantified by flow cytometry in the intestine of WT and Ahr^–/–^ mice. Data are representative of three independent experiments with *n* = 3–4 mice per group. **, P < 0.01, unpaired *t* test. **(B)** Eosinophil numbers (per tissue, per tibia, or per 1 ml blood) were assessed by flow cytometry across different tissues in *Epx*^+/+^*Ahr*^fl/fl^ and *Epx*^Cre/+^*Ahr*^fl/fl^ mice. Data are pooled from three independent experiments with *n* = 3–7 mice per genotype. *, P < 0.05, unpaired *t* test with Holm–Sidak correction for multiple testing. **(C)** Eosinophil progenitors (EoP) in the bone marrow were identified as CD45^+^lineage^–^Sca1^–^CD117^lo^CD125^+^ cells by flow cytometry. The number of eosinophil progenitors per tibia is shown. Data are pooled from two independent experiments with *n* = 3–5 mice per genotype. **(D and E)** Mice were treated with 1 mg/ml EdU in drinking water for 6 d. EdU^+^ and EdU^–^ eosinophils in the intestine were quantified by flow cytometry. Data are representative of two independent experiments with *n* = 5 mice per genotype. *, P < 0.05; **, P < 0.01, unpaired *t* test. **(F)** Schematic of adoptive transfer experiments. **(G and H)** Percentage of WT and *Ahr*^–/–^ eosinophils recovered from the colon and small intestine of recipient Δdbl*Gata1* mice at different time points after adoptive transfer. Dotted line, percentage of *Ahr*^–/–^ eosinophils at the time of injection. Data are representative of three independent experiments with *n* = 3–5 mice per time point. **, P < 0.01; ***, P < 0.001, one-sample *t* test against the injected percentage.

**Figure S4.**
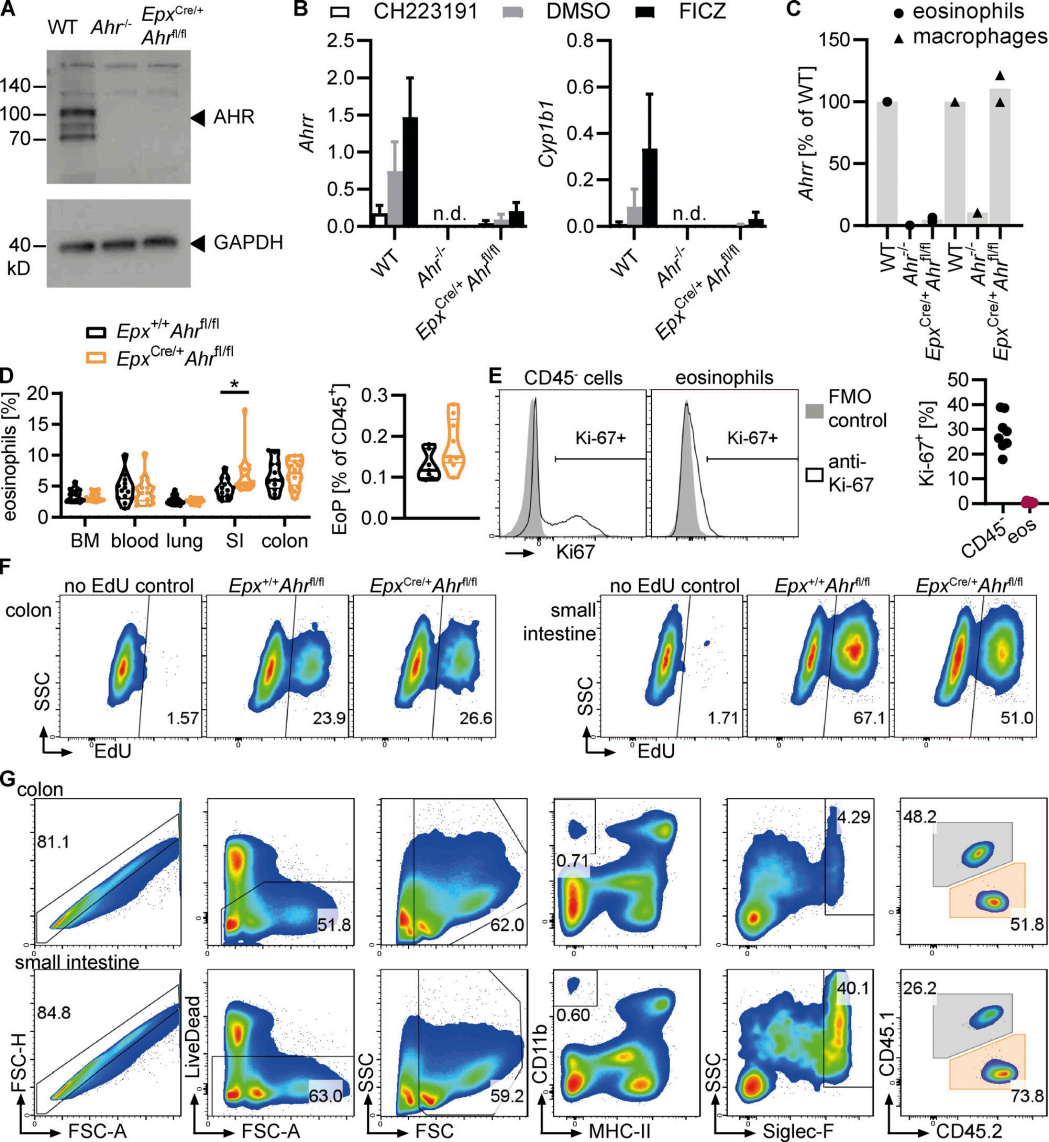
**AHR regulates eosinophil survival in the small intestine. (A)** Western blot for AHR and GAPDH in WT, *Ahr*^–/–^, and *Epx*^Cre/+^*Ahr*^fl/fl^ BMDEo from culture day 14. Data are from *n* = 1 experiment. **(B)** BMDEo from culture day 14 were stimulated for 4 h with 3 μM CH223191, 5 nM FICZ, or DMSO control. Gene expression of AHR target genes was determined by qPCR and normalized to *Hprt*. Shown is mean ± SEM of *n* = 2 experiments. **(C)** *Ahrr* expression in sorted small intestinal eosinophils and macrophages of different genotypes was determined by qPCR and normalized to *Hprt* and is expressed as a percentage of *Ahrr* expression in WT for each cell type. Data are from *n* = 1–2 mice per genotype. **(D)** Frequency (percentage of CD45^+^ cells) of eosinophils across different tissues and of eosinophil progenitors (EoP) in the bone marrow. Data are pooled from two to three independent experiments. *t* test; *, P < 0.05. **(E)** Representative flow cytometry plots and quantification of Ki-67 staining in small intestinal eosinophils and CD45^−^ cells (including epithelial and stromal cells). Less than 1% of eosinophils were Ki-67^+^. Data are from one experiment with *n* = 7 mice. FMO, fluorescence minus one. **(F)** Representative flow cytometry plots depicting EdU staining of eosinophils in the colon and small intestine after 6 d of continuous EdU administration in drinking water (1 mg/ml). **(G)** Representative flow cytometry plots depicting eosinophil gating in the colon and small intestine of Δdbl*Gata1* mice after adoptive transfer of mixed BMDEo cultures. WT and *Ahr*^–/–^ eosinophils were distinguished based on their expression of CD45.1 and CD45.2.

We next analyzed the role of AHR in eosinophils in vivo. Eosinophil numbers were increased in the small intestine but not in the colon of *Ahr*^–/–^ mice (Fig. 6 A). AHR affects multiple immune cell types, including ILC2s, which regulate eosinophil numbers (Li et al., 2018), and *Ahr*^–*/*–^ mice are more prone to intestinal inflammation (Stockinger et al., 2021). To determine whether the effect of AHR on eosinophil numbers is cell intrinsic or indirect, we generated mice with eosinophil-specific *Ahr* deletion by crossing *eosinophil peroxidase*-Cre mice with *Ahr*^fl/fl^ mice (*Epx*^Cre/+^*Ahr*^fl/fl^). We confirmed deletion of AHR in *Epx*^Cre/+^*Ahr*^fl/fl^ BMDEo by Western blot (Fig. S4 A). Moreover, these cells failed to induce *Ahrr* or *Cyp1b1* in response to FICZ stimulation (Fig. S4 B). FACS-sorted intestinal eosinophils, but not macrophages, from *Epx*^Cre/+^*Ahr*^fl/fl^ mice lacked *Ahrr* expression (Fig. S4 C), confirming efficient and specific AHR pathway deletion in eosinophils. Eosinophil number and frequency were increased in the small intestine of *Epx*^Cre/+^*Ahr*^fl/fl^ mice in comparison with *Epx*^+/+^*Ahr*^fl/fl^ control mice (Figs. 6 B and S4 D). This demonstrates that AHR controls eosinophil numbers in a cell-intrinsic manner. There was no difference in eosinophil number or frequency in any other tissue or in eosinophil progenitors (Fig. 6, B and C; and Fig. S4 D), suggesting that AHR-mediated control of eosinophil numbers is dependent on additional factors, such as the availability of ligands or the tissue microenvironment.

Tissue eosinophils are thought to be terminally differentiated cells that no longer proliferate under homeostatic conditions. We verified this by staining for Ki-67, a marker of cell proliferation. Compared with intestinal epithelial cells, which undergo continuous renewal, intestinal eosinophils did not stain for Ki-67 (Fig. S4 E). Thus, the effect of AHR on eosinophil numbers could be due to either controlling their survival or altering their migration. To distinguish between these possibilities, we administered EdU in the drinking water for 6 d and quantified the number of EdU-positive and -negative eosinophils in the small intestine and colon of *Epx*^Cre/+^*Ahr*^fl/fl^ mice and controls (Fig. S4 F). There was no significant difference between genotypes in the percentage or absolute numbers of EdU^+^ and EdU^–^ eosinophils in the colon (Fig. 6, D and E). Approximately 25% of colonic eosinophils were EdU^+^, corresponding to a half-life of 13.3 d. In the small intestine, the frequency of EdU^+^ eosinophils was significantly different between genotypes (Fig. 6 D), corresponding to a half-life of 5.9 d for *Epx*^Cre/+^*Ahr*^fl/fl^ mice and 4.2 d for *Epx*^+/+^*Ahr*^fl/fl^ controls. The absolute number of EdU^+^ cells was similar between genotypes, implying that the same number of eosinophils had migrated into the small intestine over the 6 d (Fig. 6 E). The number of EdU^–^ cells, however, was significantly higher in *Epx*^Cre/+^*Ahr*^fl/fl^ mice. This indicates that a higher number of eosinophils older than 6 d survived in the absence of AHR. To confirm these survival differences, BMDEo from WT and *Ahr*^–/–^ mice were cotransferred at a 1:1 ratio into eosinophil-deficient Δdbl*Gata1* mice (Fig. 6 F). The percentages of WT and *Ahr*^–/–^ eosinophils retrieved from recipients was then analyzed at different time points (Fig. S4 G). On day 3 after transfer, similar numbers of WT and Ahr^–/–^ eosinophils were found in the colon and small intestine of recipients (Fig. 6, G and H), indicating that migration was not affected by AHR. The percentages of WT and *Ahr*^–/–^ eosinophils remained constant in the colon. In the small intestine, however, many more *Ahr*^–/–^ than WT eosinophils were retrieved on days 6 and 9. This demonstrates that *Ahr*^–/–^ eosinophils survive longer than WT eosinophils in the small intestine. Taken together, these data confirm that AHR regulates eosinophil numbers in the small intestine by limiting their survival.

### AHR-deficient eosinophils show increased degranulation in vivo

**Figure 7.**
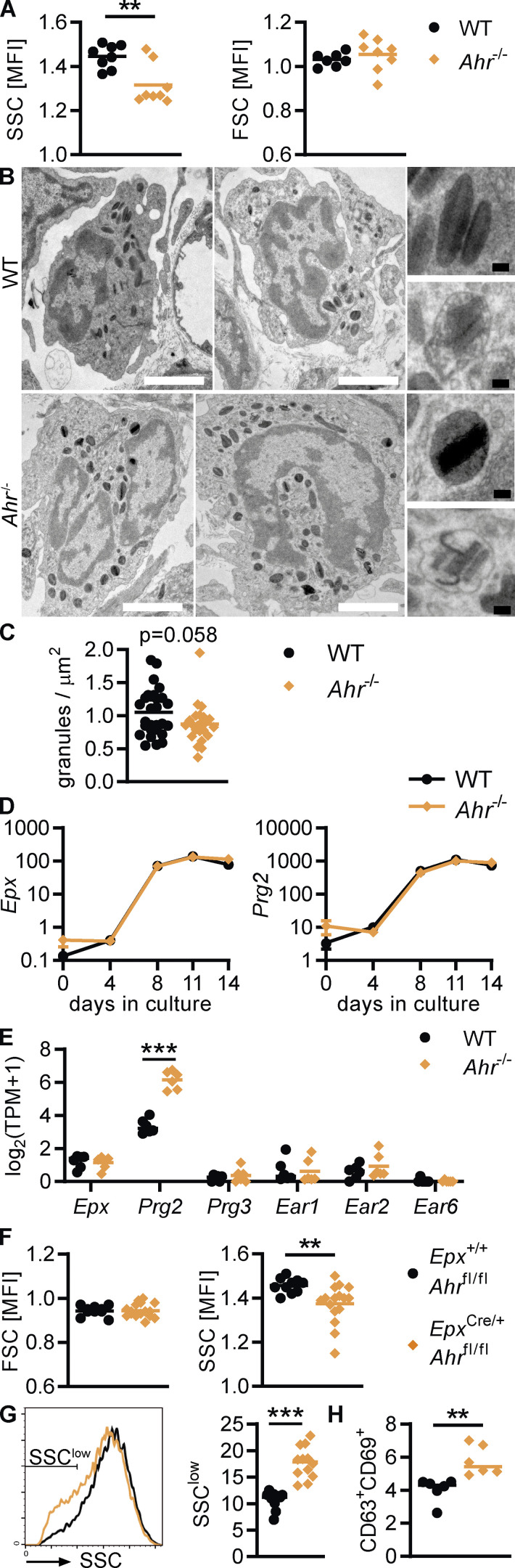
**AHR-deficient eosinophils show increased degranulation in vivo. (A)** Mean fluorescence intensity (MFI) was determined by flow cytometry. Data are pooled from two independent experiments with *n* = 4 mice per genotype each. **, P < 0.01, unpaired *t* test. **(B)** EM of the duodenum of WT and *Ahr*^–/–^ mice. Representative images show examples of eosinophils and granules. White scale bar: 2 μm, black scale bar: 100 nm. **(C)** Granules were counted in 10–15 cells/mouse on EM images, and data are pooled from two mice per genotype. The number of granules was normalized to the area of cytoplasm (cell area − nucleus area). Unpaired *t* test, P = 0.0582. **(D)** Gene expression in BMDEo at different time points in culture was determined by qPCR and normalized to *Hprt*. Shown is the mean ± SEM of four independent experiments. **(E)** RNA sequencing data of small intestinal eosinophils from *n* = 6 mice per genotype. **(F–H)** MFI and surface expression were determined by flow cytometry. Data are pooled from three independent experiments with *n* = 3–6 mice per genotype (F and G) or from one experiment (H). **, P < 0.01; ***, P < 0.001, unpaired *t* test. TPM, transcripts per million.

Eosinophils from *Ahr*^–/–^ mice showed decreased side scatter (SSC), an indication of reduced granularity, whereas the forward scatter (FSC; cell size) was unchanged (Fig. 7 A). We performed EM of the duodenal lamina propria to analyze eosinophil granules in more detail. Most granules from both WT and *Ahr*^–/–^ mice were intact, but signs of degranulation, such as loss of core or matrix, were occasionally visible (Fig. 7 B), and granule numbers per cell were on average lower in eosinophils from *Ahr*^–/–^ mice (P = 0.058; Fig. 7 C). Reduced granularity suggests either a reduction in granule formation or increased degranulation of mature eosinophils. We therefore compared eosinophil granule protein expression during development of WT and *Ahr*^–/–^ BMDEo. Eosinophils of both genotypes upregulated *Epx* and *Prg2* at the same rate (Fig. 7 D), suggesting that AHR does not control major granule protein expression. In line with this finding, intestinal eosinophils from *Ahr*^–/–^ mice showed no reduction in mRNA expression of granule proteins (Fig. 7 E). We confirmed the reduction in granularity by flow cytometry in *Epx*^Cre/+^*Ahr*^fl/fl^ mice, demonstrating that this change is due to cell-intrinsic action of AHR (Fig. 7 F). The SSC profile revealed that maximum granularity was similar between the two genotypes, while *Epx*^Cre/+^*Ahr*^fl/fl^ mice contained a higher number of SSC-low eosinophils (Fig. 7 G). We therefore assessed markers of degranulation. Translocation of CD63 to the cells surface is a sign of active degranulation, and upregulation of CD69 is associated with activation (Mahmudi-Azer et al., 2002; Nopp et al., 2000). The proportion of CD63^+^CD69^+^ eosinophils was increased in *Epx*^Cre/+^*Ahr*^fl/fl^ mice (Fig. 7 H). We conclude that AHR does not affect granule formation during eosinophil development but limits eosinophil degranulation in the small intestine. AHR-deficient eosinophils may be hyperresponsive to microbial and immunological signals, which results in increased degranulation.

### Eosinophil adhesion and ECM interactions are regulated by AHR

**Figure 8.**
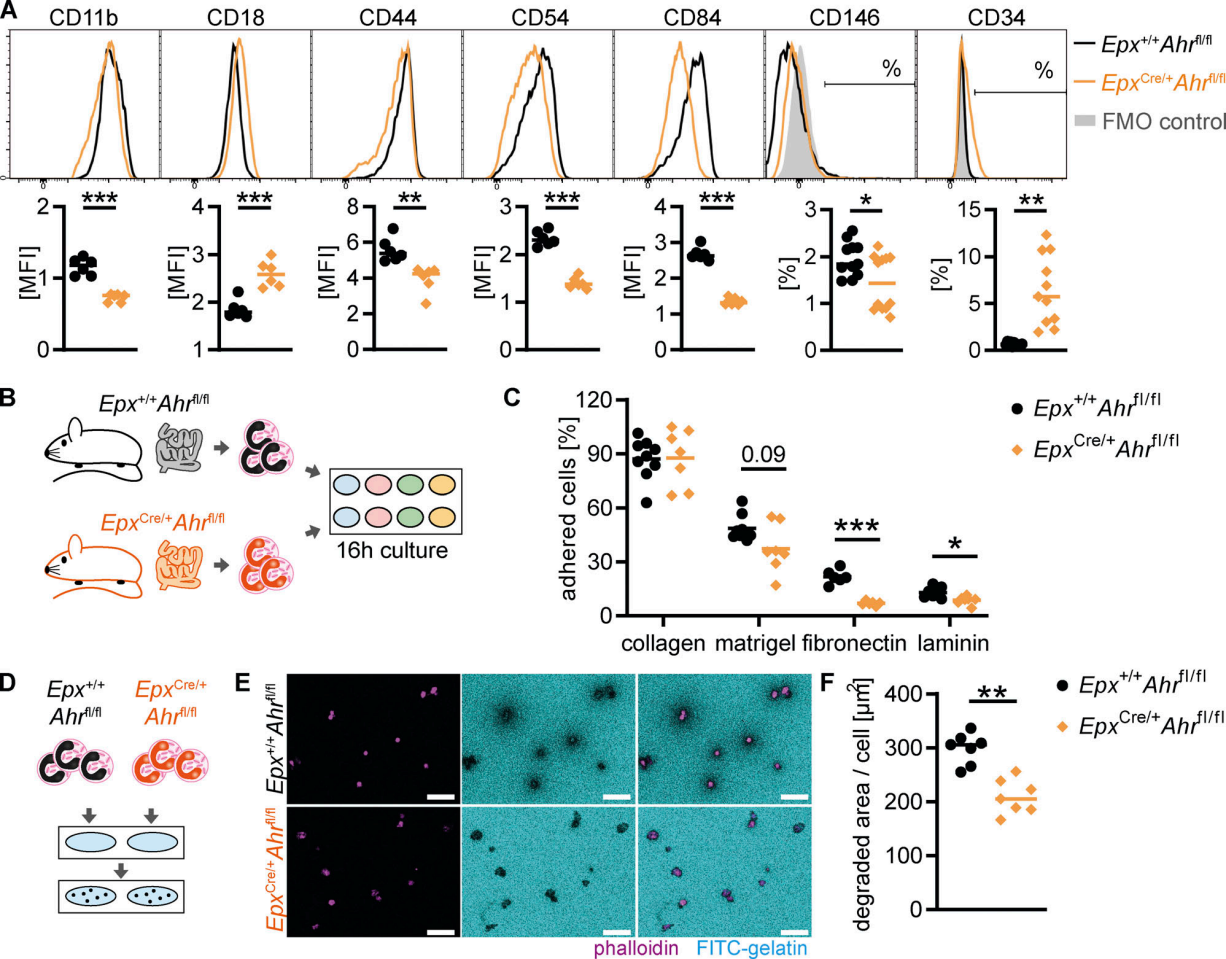
**AHR deficiency alters eosinophil–ECM interactions and remodeling. (A)** Flow cytometry of cell adhesion molecules on small intestinal eosinophils pregated as live, CD45^+^CD11b^+^MHC-II^–^SiglecF^+^SSC^hi^ cells. Representative histograms and quantification are shown. Mean fluorescence intensity (MFI; arbitrary units) is shown for CD11b, CD18, CD44, CD54, and CD84. Data are representative of two independent experiments with *n* = 6 mice per genotype and were analyzed by *t* test. **, P < 0.01; ***, P < 0.001. Percentage of positive cells is shown for CD146 and CD34. Data are pooled from two independent experiments with *n* = 6 mice per genotype and were analyzed by *t* test. *, P < 0.05; **, P < 0.01. FMO, fluorescence minus one. **(B and C)** Eosinophils were FACS-sorted from the small intestine and seeded onto wells coated with different ECM components. After 16 h, nonadherent cells were washed away, and adherent cells were quantified. **(C)** Data are pooled from two independent experiments for *n* = 6–9 mice per genotype and were analyzed by *t* test followed by Holm–Sidak correction. *, P < 0.05; ***, P < 0.001. **(D–F)** Eosinophils were FACS-sorted from the small intestine and seeded onto FITC-gelatin–coated wells. Cells were cultured for 22 h, and the area of FITC-gelatin degradation was measured on fluorescent micrographs. **(E)** Representative images. Scale bar: 50 μm. **(F)** Data are pooled from two independent experiments for *n* = 7 mice per genotype and were analyzed by *t* test. **, P < 0.01. MFI, mean fluorescence intensity.

The transcriptomic changes in AHR-deficient eosinophils suggested a reduction in adhesion receptor expression and in ECM remodeling. We sought to validate adhesion receptor expression at the protein level. Small intestinal eosinophils from *Epx*^Cre/+^*Ahr*^fl/fl^ mice expressed lower levels of the cell–cell and cell-matrix adhesion receptors CD11b, CD44, CD54, CD84, and CD146 than *Epx*^+/+^*Ahr*^fl/fl^ eosinophils, whereas expression of CD18 and CD34 were increased in *Epx*^Cre/+^*Ahr*^fl/fl^ eosinophils (Fig. 8 A). This suggests a reduced ability of AHR-deficient eosinophils to adhere to the ECM or to other cells. To determine the functional relevance of these changes, eosinophils were sorted from the small intestine and cultured on wells coated with different ECM components (Fig. 8 B). Small intestinal eosinophils adhered readily to a collagen 1 matrix (90% adhesion) and to a lesser extent to Matrigel, fibronectin, or laminin (Fig. 8 C). AHR-deficient eosinophils adhered to collagen at the same rate as controls, but adherence to Matrigel, fibronectin, and laminin was reduced, suggesting that the changes in cell adhesion receptor expression impaired their ability to interact with the ECM. Cell-matrix adhesion is a critical step in ECM degradation (Dalaka et al., 2020; Ivaska and Heino, 2000; Qi et al., 2016). Moreover, the transcriptomic data suggested that AHR-deficient eosinophils have a reduced ability to remodel the ECM. To test this directly, small intestinal eosinophils were seeded onto wells coated with fluorescein-coupled gelatin, and the extent of gelatin degradation was determined after 22 h (Fig. 8 D). Gelatin degradation at the site of eosinophil adhesion was readily detectable for cells from both genotypes; however, the area of degradation per cell was much smaller for *Epx*^Cre/+^*Ahr*^fl/fl^ eosinophils (Fig. 8, E and F). These results demonstrate that AHR controls how eosinophils adhere to and remodel the ECM in the small intestine. Although eosinophils have long been recognized for their involvement in wound healing and fibrosis, our data show direct evidence of their ability to degrade the ECM.

### AHR deficiency in eosinophils modulates the intestinal immune system

**Figure 9.**
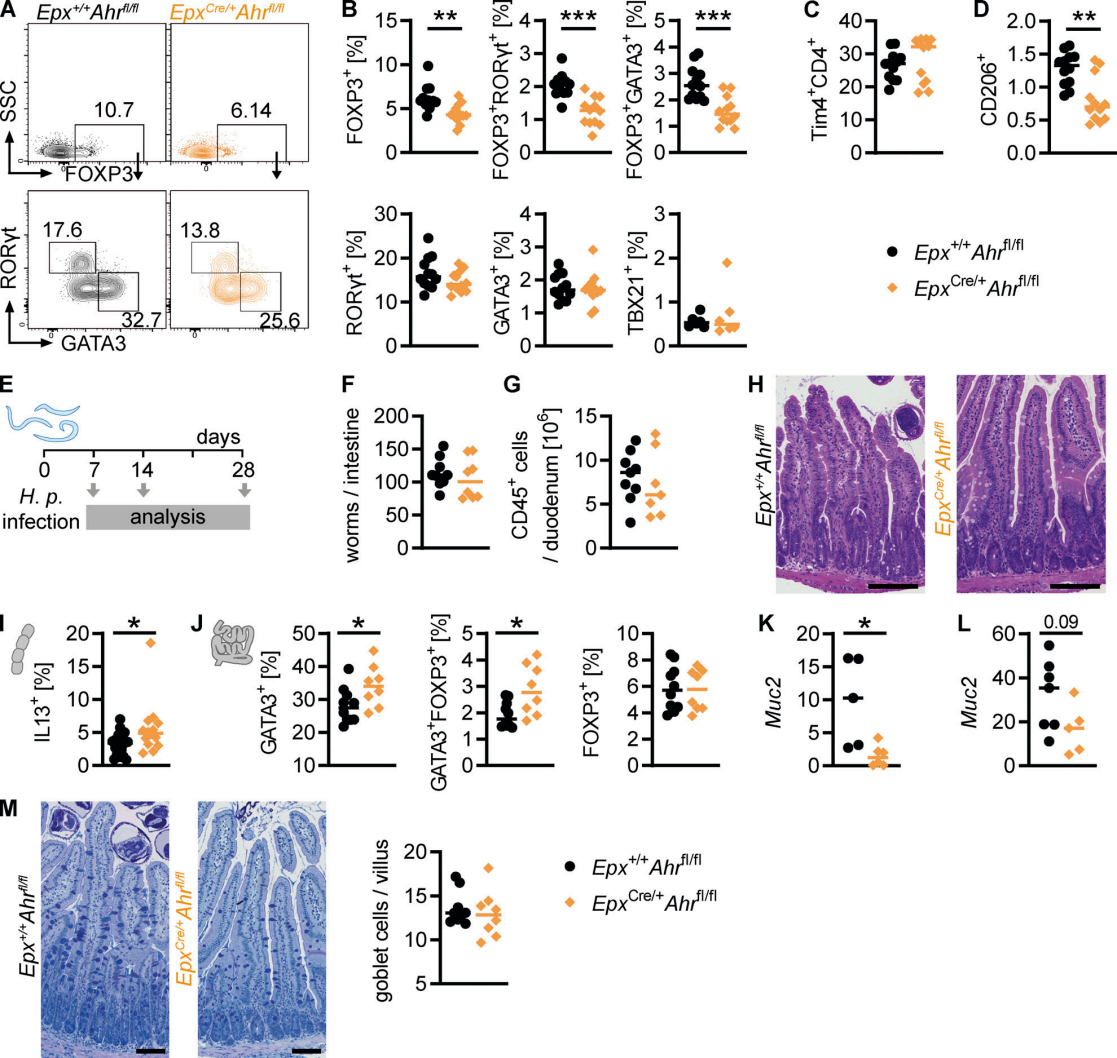
**AHR deficiency in eosinophils modulates the intestinal immune system. (A and B)** Small intestinal CD45^+^CD3^+^CD4^+^TCRb^+^ T cells were analyzed by flow cytometry. **(A)** Representative flow cytometry plots. **(B)** Frequency of different T helper cell populations. **(C and D)** Flow cytometry of small intestinal macrophages (CD45^+^CD11b^+^CD64^+^MHC-II^+^). In A–D, data are pooled from two independent experiments for a total of *n* = 12 mice per genotype and were analyzed by *t* test. **, P < 0.01; ***, P < 0.001. **(E–M)** *Epx*^+/+^*Ahr*^fl/fl^ and *Epx*^Cre/+^*Ahr*^fl/fl^ mice were infected with 200 *H.p.* L3 larvae on day 0 and analyzed 7, 14 or 28 d after infection. **(F)** Worm burden was quantified at day 14 after infection. **(G)** CD45^+^ cells in the duodenum were quantified by flow cytometry. **(H)** Representative images of H&E-stained duodenal sections from day 14 after infection. **(I)** Cytokine expression in CD4^+^ T cells from the mesenteric lymph node on day 14 after infection. **(J)** Transcription factor staining of lamina propria CD4^+^ T cells on day 14 after infection. **(K and L)** Gene expression was determined by qPCR and normalized to *Hprt* at day 7 (K) and day 14 (L) after infection. **(M)** Representative images and goblet cell quantification of AB-PAS–stained duodenal sections from day 14 after infection. *, P < 0.05; unpaired t test (I–L). In H and M, black scale bars: 0.1 mm.

**Figure S5.**
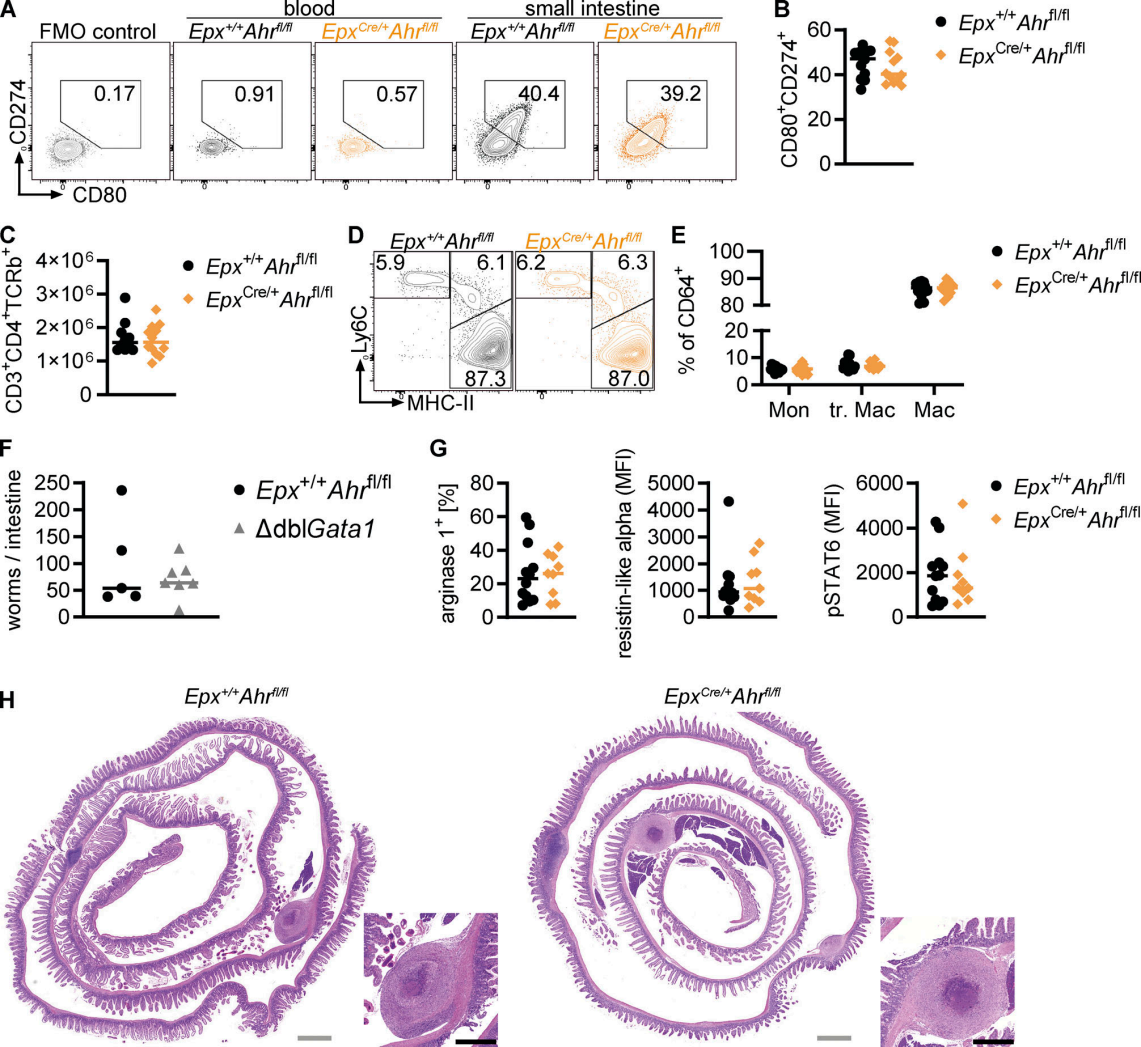
**AHR deficiency in eosinophils modulates the intestinal immune system. (A and B)** Representative flow cytometry plots and quantification of CD80^+^CD274^+^ eosinophils in the blood and small intestine. FMO, fluorescence minus one. **(C)** Small intestinal CD45^+^CD3^+^CD4^+^TCRb^+^ T cells were analyzed by flow cytometry. The total cell number per small intestine is shown. **(D and E)** Flow cytometry of the small intestinal monocyte-macrophage compartment. Cells were pregated as live CD45^+^CD11b^+^CD64^+^ cells. In A–E, data are pooled from two independent experiments for a total of *n* = 12 mice per genotype and were analyzed by *t* test. **(F)** Worm burden was quantified in the small intestine at day 7 after *H.p.* infection. Data are from one experiment with *n* = 5–7 mice per genotype. **(G)** Expression of alternative activation markers in duodenal macrophages on day 14 after *H.p.* infection. Data are pooled from two independent experiments. MFI, mean fluorescence intensity. **(H)** Representative images of H&E-stained duodenum on day 28 after *H.p.* infection. Gray scale bars: 1 mm, black scale bars: 0.5 mm.

We wondered whether AHR deficiency in eosinophils would have wider effects on the intestinal immune system. Several studies have linked eosinophils to T helper cell differentiation and activity, suggesting they promote regulatory T cell differentiation (Chen et al., 2015) or suppress Th1 and Th17 cells (Arnold et al., 2018; Sugawara et al., 2016). T helper cell subsets in the small intestine were analyzed by flow cytometry, revealing a reduction in regulatory FOXP3^+^ cells and FOXP3^+^GATA3^+^ and FOXP3^+^RORγt^+^ cells in *Epx*^Cre/+^*Ahr*^fl/fl^ mice (Fig. 9, A and B). This suggests that AHR-deficient eosinophils have a diminished ability to promote regulatory T cell differentiation. A previous study found that eosinophils suppress Th1 cells through PD-L1 (CD274; Arnold et al., 2018), and a recent preprint paper described a subset of CD80^+^CD274^+^ eosinophils with high *Ahr* expression (Gurtner et al., 2021 *Preprint*). We found that this CD80^+^CD724^+^ eosinophil subset was not affected by AHR deficiency (Fig. S5, A and B), and the frequencies and absolute numbers of total CD4^+^ T cells and Th1, Th17, and Th2 cells were unchanged (Figs. S5 C and 9 B). In the fat tissue, eosinophils have been shown to maintain macrophage populations (Wu et al., 2011). The monocyte-to-macrophage differentiation in the small intestine was not affected in *Epx*^Cre/+^*Ahr*^fl/fl^ mice (Fig. S5, D and E). Tissue-resident Tim4^+^CD4^+^ macrophages were present at the same frequency in *Epx*^Cre/+^*Ahr*^fl/fl^ mice and *Epx*^Cre/+^*Ahr*^fl/fl^ controls (Fig. 9 C), but there was a small reduction in the frequency of CD206^+^ macrophages in the small intestine of *Epx*^Cre/+^*Ahr*^fl/fl^ mice (Fig. 9 D). Eosinophils have been shown to promote CD206 expression on macrophages (Toor et al., 2020), and this function seems to be impaired in *Epx*^Cre/+^*Ahr*^fl/fl^ eosinophils. These findings show that AHR deficiency in eosinophils has broad effects on the intestinal immune system.

To determine whether the functional changes in AHR-deficient eosinophils influenced the response to an immunological challenge, we chose *Heligmosomoides polygyrus* (*H*.*p*.) infection, a model of small intestinal inflammation and tissue damage (Fig. 9 E). *Epx*^Cre/+^*Ahr*^fl/fl^ mice and *Epx*^+/+^*Ahr*^fl/fl^ controls had the same worm burden on days 7 and 14 after infection (not depicted and Fig. 9 F). This was unsurprising, as eosinophil-deficient Δdbl*Gata1* mice have no change in worm burden after infection (Fig. S5 F; Strandmark et al., 2017). Overall levels of inflammation were also similar between the two genotypes (Fig. 9, G and H). We analyzed the infiltrating immune cells in more detail and found increased expression of IL-13 by CD4^+^ T cells in the mesenteric lymph nodes and increased GATA3^+^ and GATA3^+^FoxP3^+^ Th2 cells in the lamina propria of infected *Epx*^Cre/+^*Ahr*^fl/fl^ mice (Fig. 9, I and J). It has previously been reported that eosinophils suppress Th2 responses during *H.p.* infection (Strandmark et al., 2017), and this ability seems to be limited in AHR-deficient eosinophils. FOXP3^+^ regulatory T cells were not different between *Epx*^Cre/+^*Ahr*^fl/fl^ mice and controls after infection (Fig. 9 J). Helminth-derived products can directly induce regulatory T cell differentiation during infection (Grainger et al., 2010; Johnston et al., 2017), which may have masked the effect of eosinophils. We analyzed myeloid cells to determine if this increased Th2 response led to an increase in alternatively activated macrophages but found no obvious differences in the expression of arginase 1, resistin-like α, or phospho-STAT6 in macrophages (Fig. S5 G). There was a slight decrease in the expression of the goblet cell marker *Muc2* in *Epx*^Cre/+^*Ahr*^fl/fl^ mice, but no noticeable reduction in the number of goblet cells (Fig. 9, K–M). On day 28 after infection, granulomas formed in mice of both genotypes, and no obvious histological differences were observed (Fig. S5 H). Taken together, these data show that AHR in eosinophils alters the intestinal T helper cell compartment, although it is unclear how this affects the immune response to *H.p.* infection.

## Discussion

The presence of large numbers of eosinophils in the healthy intestine suggests they carry out physiological functions and can adjust to the local environment (Lee et al., 2010; Shah et al., 2020). Here, we show that eosinophils profoundly change their transcriptome from the bone marrow to the small intestine, highlighting for the first time that eosinophils undergo tissue adaptation. Although some level of heterogeneity between eosinophils from different tissues has long been recognized, particularly in their expression of surface markers (Masterson et al., 2021), the concept of tissue adaptation as such has not been explored for eosinophils. More recent publications have described subsets of eosinophils within the intestine and lung (Mesnil et al., 2016; Xenakis et al., 2018), but eosinophils were not directly compared between different tissues.

We demonstrated that AHR controls part of the transcriptomic changes that eosinophils undergo in the intestine. The high availability of AHR ligands in the small intestine and the high expression of AHR specifically in intestinal eosinophils allow for a tissue-specific regulation of AHR-mediated transcriptional programs and show how local environmental imprinting can affect cell identity and function. Additional factors are likely at play that regulate the remaining genes that are changed between bone marrow and small intestinal eosinophils. They may include other transcription factors that are highly upregulated in the small intestine (but were not differentially expressed between *Ahr*^–/–^ and WT eosinophils), such as the AP-1 family, which can regulate large-scale chromatin remodeling (Bejjani et al., 2019). Cytokines and other mediators abundant in the intestinal microenvironment likely also play a role. For example, TGFb1 and IL-10 have been shown to control intestinal adaptation of macrophages (Schridde et al., 2017; Zigmond et al., 2014) and might be a factor for eosinophils as well.

As the role of intestinal eosinophils remains largely unknown, our dataset reveals several likely functions, including in the remodeling of the ECM; in antimicrobial defense; as producers of chemokines, cytokines, and lipid mediators; and as highly interactive cells. Intestinal eosinophils downregulated genes associated with transcription and translation, supporting the idea that they preform and store molecules for later use while in the bone marrow. It also suggests that those genes that are upregulated in the intestine are acutely needed to perform important eosinophil functions. Intestinal eosinophils upregulated ECM components such as collagens and laminins, as well as matrix metalloproteases. This indicates that eosinophils remodel the ECM, including the basement membrane, and is in line with a recent preprint article that used proteomics to find substantial changes of the intestinal ECM composition in the absence of eosinophils (Shah et al., 2021 *Preprint*). Several studies found that eosinophils affect the intestinal barrier (Buonomo et al., 2016; Johnson et al., 2015; Singh et al., 2019). We found that eosinophils induce expression of many antimicrobial and barrier maintenance factors such as the trefoil factor family, *Reg3b*/*Reg3g*, and α-defensins, offering a possible mechanism by which they maintain the intestinal barrier. Eosinophils have not previously been recognized as producers of trefoil factor family and *Reg3b*/*Reg3g* proteins, which are thought to be mostly expressed by intestinal epithelial cells (Hoffmann, 2004; Shin and Seeley, 2019).

It is perhaps not surprising that intestinal eosinophils upregulated many genes involved in cell–cell communication and cell–cell junction formation. They were previously shown to interact with other immune cells such as T helper cells and dendritic cells (Arnold et al., 2018; Chu et al., 2014a; Sugawara et al., 2016), mostly through cytokines but also via direct contact. Our data suggest that eosinophils are highly interactive and form close contact with other cells in the lamina propria. Whether this is mostly directed at other immune cells or stromal cells is not clear. Many open questions remain, including whether intestinal eosinophils are more tolerant than those from other tissues (as is generally thought to be the case for gut-resident immune cells; Bain and Mowat, 2014; Faria et al., 2017). It is also unclear how special intestinal eosinophils are compared with those from other tissues such as lung, thymus, or uterus.

AHR controlled part of the gene expression program of intestinal eosinophils. AHR-regulated functions overlapped with pathways upregulated in intestinal eosinophils, including cell junctions, ECM remodeling, and antimicrobial peptides, indicating that these important intestinal functions are at least in part regulated by AHR. We validated these transcriptomic data on a functional level, showing that AHR regulates cell adhesion receptor expression, eosinophil–ECM adhesion, and gelatin degradation. This demonstrated a key function of intestinal eosinophils to remodel the ECM.

Our findings raise the question whether AHR may influence intestinal tissue adaptation of other immune cells. Previous work has shown that the AHR pathway is active in intestinal T cell subsets (Li et al., 2011), innate lymphoid cells (Li et al., 2018; Qiu et al., 2012), and myeloid intestinal cell types such as macrophages and dendritic cells (Brandstatter et al., 2016). We here confirmed these findings in AHR reporter mice and by qPCR. Moreover, we found that most intestinal cell types express AHR to some degree (or in subpopulations), suggesting that its role in mediating adaptation to the intestine might be more general. AHR reporter mice reveal widespread AHR expression in the immune as well as stromal compartments.

The increased survival of AHR-deficient small intestinal eosinophils is in apparent contrast to the effect of AHR on ILC3s and intraepithelial lymphoid cells, where it is required for survival (Cervantes-Barragan et al., 2017; Gomez de Agüero et al., 2016). It is possible that increased activation of NF-κB and the MAPK pathway in the absence of AHR may prolong the survival of eosinophils (Kankaanranta et al., 1999; Schwartz et al., 2015). We did not fully explore how AHR interacts with the range of signals and pathways that have been shown to regulate eosinophil survival (Masterson et al., 2021; Park and Bochner, 2010; Shen and Malter, 2015) and cannot rule out that additional mechanisms are involved. For example, ST18 was the most upregulated transcription factor in WT versus *Ahr*^–/–^ eosinophils. There are no studies on ST18 function in eosinophils, but it has been shown to upregulate proapoptotic genes in fibroblasts (Yang et al., 2008). AHR suppressed ST18 and may thereby increase eosinophil life span. The effect of AHR deficiency on retaining a more progenitor-like state in cells may also be linked to increased survival of *Ahr*^–/–^ eosinophils. Our data show that colonic eosinophils survived much longer than those in the small intestine. This has not been previously reported and is longer than the half-life of any other tissue eosinophil studied so far (Carlens et al., 2009). Colonic eosinophils express AHR, suggesting that a difference in ligand availability or other microenvironmental factors may be the reason for the differences in survival.

AHR has been proposed to play an anti-inflammatory role in myeloid cells, mostly based on studies with LPS-treated mice or cell cultures (Kimura et al., 2009; Nguyen et al., 2010; Thatcher et al., 2016). The role of AHR in intestinal eosinophils appears to be more complicated, as the lack of AHR led to increased expression of some cytokines (*Il1b* and *Il4*) but decreased expression of others (*Csf1* and *Cxcl3*). Moreover, AHR regulated numerous pathways in eosinophils that are not related to inflammation, such as triglyceride metabolism and protein translation and pathways mentioned above such as ECM remodeling and tight junctions. Nevertheless, AHR-deficient eosinophils increased pathways relating to inflammatory bowel disease, TNFR2, and NF-κB pathways. In addition, we found that AHR-deficient eosinophils degranulate more readily in the small intestine. This suggests that AHR dampens eosinophil inflammatory responses, but a more detailed understanding of eosinophil function in the context of the intestinal environment is needed to resolve this question.

AHR deficiency in eosinophils had wider effects on the intestinal immune system, in particular on the T helper cell compartment. Regulatory T cells were reduced in the absence of AHR on eosinophils. In the context of *H.p.* infection, Th2 cells were increased in the absence of AHR. It is possible that overactive AHR-deficient eosinophils drove a stronger Th2 response after being exposed to helminth products or that they failed to suppress Th2 cells, as was previously suggested (Strandmark et al., 2017).

Unbiased transcriptional profiling was conducted here to shed light on potential functions of intestinal eosinophils. This is the first publicly available bulk RNA-sequencing dataset of small intestinal eosinophils. We used multiple databases containing gene ontology and pathway information to obtain insight into intestinal eosinophils and AHR-dependent functions based on the gene expression data. However, the lack of eosinophil-specific pathways in these databases poses a limit on the interpretation of these results and highlights the need for more functional eosinophil transcriptome data. Eosinophils express a range of RNases (Cormier et al., 2001; Rosenberg and Domachowske, 2001), similar to pancreatic tissue (Augereau et al., 2016), which makes it difficult to conduct RNA sequencing. In our hands, many samples could not be used for sequencing because of insufficient RNA quality. This is also a possible reason for the relative paucity of RNA-sequencing data on eosinophils to date, and why some studies report RNA sequencing data with *n* = 1 replicate (Reichman et al., 2017) or high variability where RNA quality was not reported (Arnold et al., 2018).

In summary, our study demonstrates that eosinophils undergo tissue adaptation in the small intestine, changing their function and longevity, and this process is in part controlled by AHR. This opens up the field to reveal the full extent of eosinophil adaptation across all tissues in which they reside under homeostasis. Such future studies will improve our understanding of the distinct functions of tissue-resident eosinophils.

## Materials and methods

### Human blood donors

Peripheral blood samples were collected by a trained phlebotomist from consenting healthy adult volunteers. This study was approved by the Crick’s Human Ethics Group and conducted under HTA license number 12650.

### Human eosinophil isolation and culture

Blood samples (40 ml) were drawn into EDTA tubes. The blood was layered over Histopaque 1.077 and 1.119 (Sigma-Aldrich) and centrifuged at 800 *g* for 30 min at room temperature (RT). The mononuclear (peripheral blood mononuclear cells [PBMCs]) and granulocyte layers were isolated and washed, and remaining erythrocytes were lysed with ammonium-chloride-potassium (ACK) lysis buffer. Eosinophils were purified using the EasySep Human Eosinophil Isolation Kit (StemCell Technologies) according to manufacturer’s instructions. To determine eosinophil purity, cells were stained with antibodies (Table 1) as described below and analyzed by flow cytometry. Eosinophils were identified as live, Siglec8^+^EMR1^+^ cells. Eosinophil purity ranged from 61.0 to 93.2% (average 82.4 ± 13%). Human eosinophils were cultured in RPMI 1640 (#61870-010; Thermo Fisher Scientific) with penicillin/streptomycin (#P4333; Sigma-Aldrich) and 10 ng/ml IL5 (#205-IL-005; R&D Systems) at 75,000 cells per well in duplicate and stimulated for 4 h with DMSO, 5 nM FICZ, or 3 µM CH223191.

**Table 1.** Human eosinophil antibodies

Antigen	Fluorophore	Manufacturer	Clone	Catalog number
CD14	FITC	Thermo Fisher Scientific	MEM-18	MA1-19561
CD69	PE	ImmunoTool	TP1.55.3	21330694
Siglec8	Pe/Cy7	BioLegend	7C9	347111
Siglec8 isotype control	Pe/Cy7	BioLegend	MOPC-21	400125
EMR1	A647	Bio-Rad	A10	MCA2674A647
EMR1 isotype control	A647	BioLegend	HTK888	400924
CD16	A700	BioLegend	3G8	302026
L/D	[InfraRed]	Thermo Fisher Scientific		L10119

### Mice

All mice were bred and maintained in individually ventilated cages at the Francis Crick Institute, under specific pathogen–free conditions according to the protocols approved by the UK Home Office and the ethics committee (AWERP) of the Francis Crick Institute. In this study, WT, *Ahr*^–/–^, Δdbl*Gata1*, *Epx*^+/+^*Ahr*^fl/fl^, and *Epx*^Cre/+^*Ahr*^fl/fl^ mice were used. *Ahr*^fl/fl^ mice were derived from ES cells generated on a B6 (high-affinity AHR) background by the Knockout Mouse Project (*Ahr*^*tm1c(KOMP)Stck*^). All mice were on the C57BL/6 background. All mouse strains were first re-derived via embryo or sperm transfer into the Francis Crick Institute breeding facility and bred in the same breeding facility at the Francis Crick Institute; none were directly purchased from external vendors. Both male and female mice 6–14 wk of age were used in experiments. Within experiments, mice were sex- and age-matched within 3 wk. Littermates were used for experiments with *Epx*^Cre/+^*Ahr*^fl/fl^ mice and *Epx*^+/+^*Ahr*^fl/fl^ controls, and mice were cohoused from weaning and throughout the experiment. *Ahr*^–/–^ and WT controls were not littermates and were not cohoused.

### Generation of Ahr-TdTomato mice

A C-terminal knock-in of tdTomato into the *Ahr* gene was created via CRISPR/Cas9-mediated genome editing of mouse embryonic stem (mES) cells. Briefly, a DNA donor repair template construct was designed containing a 1-kb homology arm complementary to the 3′ terminus of the *Ahr* open reading frame, followed by a P2A sequence encoding a self-cleaving peptide, the tdTomato sequence, and a 1-kb homology arm complementary to the *Ahr* 3′ UTR. A sgRNA sequence (5′-TGT​TCT​CAG​GTG​CAG​AGT​TG-3′ PAM: AGG) was selected to target 1 bp upstream of the intended insertion site immediately before the stop codon of *Ahr* exon 11 (GRCm38/mm10 chr12: 35550690). The sgRNA was cloned into the px459 V2.0 (Addgene 62988) plasmid and cotransfected with a commercially synthesized (GeneArt) 3.5-kb DNA donor repair template construct into C57Bl6 ES cells (an in-house–generated mES cell line termed B6N6.0, derived from C57BL/6N mES cells) using Lipofectamine 2000 (Thermo Fisher Scientific). Puromycin was applied for 48 h to select positively transfected cells, and after 9 d, monoclonal mES cell colonies were isolated into a 96-well plate before expansion and screening. Clones that were positive by PCR for the insertion of tdTomato sequence underwent further analysis to ensure *Ahr*-specific integration of the DNA donor repair template, by using primer sequences extending across the homology arms from the WT *Ahr* locus into tdTomato. Next, a long-range PCR product across the entire construct was sequenced to ensure clean integration of the DNA donor repair template. Successful clones were further checked to ensure the absence of plasmid DNA backbone integration by a multiplex PCR approach, and droplet digital PCR was used to confirm integration of tdTomato at the anticipated copy number. Identified clones were expanded and underwent confirmatory steps before microinjection. The selected clone was injected into 54 blastocysts and resulted in the birth of 19 offspring, including 12 male chimeras. The highest-contribution chimeras were bred to achieve germline transmission as confirmed by PCR analysis, and mice bearing the correct allele were maintained.

### *H.p.* infection

Mice were infected with 200 *H*.*p*. L3 larvae in 200 μl PBS by oral gavage on day 0. Mice were analyzed on day 7, 14, or 28 after infection.

### EdU administration and calculation of eosinophil half-life

Mice received 1 mg/ml EdU (Sigma-Aldrich) in drinking water for 3 or 6 d. EdU was replaced every 3 d. Eosinophil half-life (*t*_1/2_) in the small intestine and colon was calculated as *t*_1/2_ = *t*/log_0.5_(*N*_*t*_/*N*_0_), where *t* is the time of EdU administration (6 d), *N*_*t*_ is the percentage of EdU^–^ cells after 6 d, and *N*_0_ is the percentage of EdU^–^ cells at time 0 (100%).

### BMDEo

BMDEo were cultured as previously described (Dyer et al., 2008). In brief, bone marrow was isolated as described above under a laminar flow hood, and cells were seeded at 2 × 10^6^ cells/ml in BMDEo medium: RPMI 1640 (#10-040-CV; Corning) supplemented with 20% FCS, penicillin/streptomycin, 2 mM L-glutamine, 25 mM Hepes, nonessential amino acids, 1 mM sodium pyruvate, and 55 nM 2-mercaptoethanol. For the first 4 d, 100 ng/ml stem cell factor and 100 ng/ml FLT3L (both Peprotech) were added to the medium. Medium was changed on days 4, 8, and 11 to fresh medium with 10 ng/ml IL-5 (R&D Systems), and cell concentrations were readjusted on days 8 and 11 to 1 × 10^6^ cells/ml. On day 8, cells were transferred to a new flask. Cells were cultured at 37°C and 5% CO_2_.

### Adoptive transfer of BMDEo

BMDEo from *Ahr*^–/–^ CD45.2/.2 mice and WT CD45.1/.2 mice were mixed and cultured to day 14 as described above. Cells were gently washed three times in 50 ml PBS. Approximately 12–15 million eosinophils in 200 μl PBS were intravenously injected into the tail vein of recipient Δdbl*Gata1* mice.

### EM

Mice were terminally anesthetized and perfused with 30–50 ml of 0.1 M phosphate buffer (PB) containing 4% paraformaldehyde and 2.5% glutaraldehyde at pH 7.4 and fixed for an additional 1 h at RT in the same fixation buffer. Pieces of upper jejunal tissue were excised and transferred to PB. Samples were embedded in 2% low-melting-point agarose (A4018-50G; Sigma-Aldrich) in 0.1 M PB, and 100 µm sections were collected using a vibrating knife ultramicrotome (VT1200S; Leica Microsystems), using a speed of 1 mm/s and an amplitude of 0.75 mm. Excess agarose was removed from the sections, and tissue was stored in a 24-well plate in 0.1 M PB. Sections were postfixed in 4% paraformaldehyde and 2.5% glutaraldehyde in 0.1 M PB at a pH 7.4 for 30 min, followed by washes in 0.1 M PB (2 × 10 min), and postfixed in 1% reduced osmium (1% osmium tetroxide/1.5% potassium ferricyanide) for 60 min at 4°C. Tissue was washed (3 × 5 min in 0.1 M PB) and incubated in 1% tannic acid in 0.05 M PB for 45 min at RT, followed by quenching in 1% sodium sulfate in 0.05 M PB for 5 min at RT. After washing (3 × 5 min in dH_2_O), sections were dehydrated using a graded series of ethanol (20, 50, 75, 90, and 100% 2×, 10 min each) followed by infiltration with TAAB Epon 812 (T004; TAAB; 75:25 ethanol/Epon 2 h, 50:50 ethanol/Epon 2 h, 25:75 ethanol/Epon 2 h, and 100% Epon overnight). Sections were then flat embedded using Aclar (L4458; Agar Scientific) and polymerized at 60°C for 48 h. Ultrathin sections were cut from blocks using a 3-mm ultra 45° diamond knife (DiATOME) on an ultramicrotome (EM UC7; Leica Microsystems), collected onto formvar-coated slot grids, and poststained with lead citrate for 5 min. Sections were viewed using a transmission electron microscope (Tecnai G2 Spirit BioTwin; Thermo Fisher Scientific) at 120 kV, and images were captured using a charge-coupled device camera (Orius; Gatan).

### Ethoxyresorufin-*O*-deethylase assay

BMDEo from day 14 of culture were seeded at 1 × 10^6^ cells per 300 μl in 96-well plates and cultured with the indicated concentration of FICZ for 4 h. Cells were washed in PBS and resuspended in 100 μl PB (50 mM NaHPO_4_ and 50 mM NaH_2_PO_4_, pH 8.0) with 2 µM resorufin ethyl ether (#E3763; Sigma-Aldrich) and incubated for 30 min. Resorufin ethyl ether is converted to resorufin by the CYP1A1 enzyme. The reaction was stopped by addition of 75 μl acetonitrile containing fluorescamine (150 µg/ml). Resorufin fluorescence was measured at 535-nm excitation and 590-nm emission. Fluorescamine fluorescence was measured at 390-nm excitation and 485-nm emission. Serial dilutions of resorufin (#424455; Sigma-Aldrich) and BSA were measured in parallel to generate standard curves. Resorufin concentration was normalized to protein content.

### Western blot

BMDEo from culture day 14 were centrifuged and resuspended in lysis buffer (50 mM Tris, pH 8.0, 150 mM NaCl, 10% glycerol, and 1% NP-40) with Roche cOmplete Protease Inhibitor Cocktail and incubated at 4°C for 20 min. Nucleic acids were digested by addition of benzonase (#E1014; Sigma-Aldrich) and further incubation for 10 min at 37°C. After centrifugation at 17,000 *g* for 10 min at 4°C, the supernatant was transferred to a new tube, and the protein concentration was determined using Bradford assay. Samples were adjusted to a protein concentration of 1 µg/μl in Laemmli buffer with 10% β-mercaptoethanol and denatured for 5 min at 95°C. 15 µg protein was used per sample and resolved on 4–15% Mini-PROTEAN TGX Precast Protein Gels (#4561085; Bio-Rad) and transferred on a Trans-Blot Turbo Midi PVDF membrane (Bio-Rad) via semidry transfer. Membranes were washed, blocked in 1% skim milk, and incubated with polyclonal rabbit anti-AHR (#BML-SA21; Enzo), 1:2,000 in blocking buffer overnight at 4°C. The membrane was washed in Tris-buffered saline with 0.1% Tween 20, incubated with goat anti-rabbit HRP antibody (Thermo Fisher Scientific), and developed using ECL Plus reagent (Thermo Fisher Scientific). As a loading control, membranes were incubated with anti-GAPDH (#2118; Cell Signal) and developed as above with ECL reagent onto a photo film.

### Generation of single-cell suspensions from different tissues

Blood was drawn from the heart of terminally anesthetized mice into PBS with 50 U/ml heparin. Anticoagulated blood was layered over Histopaque 1.119 and centrifuged for 30 min, 500 *g* at RT. The leukocyte layer was washed in PBS, and remaining erythrocytes were lysed with ACK lysing buffer for 2 min at RT before washing in PBS. For lung cell isolation, mice were perfused with 5 ml PBS through the right ventricle before excising the lungs. Lung tissue was minced and digested for 40 min at 37°C, 200 rpm in HBSS, 5% FCS, 1.5 mg/ml Collagenase VIII, and 80 µg/ml DNase I. Cells were centrifuged, followed by ACK lysis, washing in PBS, and filtration through a 70-µm filter. Unless otherwise noted, cells were centrifuged at 300 *g* for 5 min at 4°C. Bone marrow was flushed from the femur and tibia with PBS, red blood cells were lysed with ACK lysing buffer, and cells were washed in PBS and filtered through 70-µm filters. Cells from spleen and thymus were isolated by mashing through a 70-µm filter, followed by ACK lysis, washing in PBS, and filtration through a 70-µm filter. Intestinal lamina propria cells were isolated by first cleaning the intestines of fecal content. Intestines were cut longitudinally and washed in PBS. and the epithelial layer was removed by incubating for 40 min at 37°C, 200 rpm in HBSS, 5% FCS, and 2 mM EDTA. Intestines were washed in PBS, minced, and digested for 25–30 min at 37°C, 200 rpm in HBSS, 5% FCS, 1.5 mg/ml Collagenase VIII, and 80 µg/ml DNase I. Cells were filtered through a 100-µm filter, washed in FACS buffer, filtered through a 70-µm filter, and washed again.

### Flow cytometry

Single-cell suspensions were prepared as described above, incubated with anti-CD16/32 (eBioscience) and fixable Live/Dead cell stain (Thermo Fisher Scientific) for 30 min at 4°C, and washed in PBS. For EdU labelling experiments, cells were fixed and stained with the Click-iT EdU Alexa Fluor 488 Flow Cytometry Assay Kit (Thermo Fisher Scientific) according to the manufacturer’s instructions. Cells were then incubated in FACS buffer with directly conjugated antibodies for 30 min at 4°C. Cells were washed in FACS buffer and optionally fixed in 3% paraformaldehyde in PBS for 30 min to 18 h at 4°C. To determine cytokine production by T cells, single-cell suspensions were incubated for 3.5 h at 37°C in culture medium (IMDM, 5% FCS, and penicillin/streptomycin) with 20 ng/ml PMA, 1 µM ionomycin, and 10 µg/ml Brefeldin A. For intracellular staining for transcription factors or cytokines, cells were fixed and permeabilized using the Transcription Factor Staining Buffer Set (#00-5523-00; eBioscience) according to the supplied protocol. For intracellular staining against pSTAT6, RETLNA, and ARG1, samples were fixed in methanol-containing Phosflow Buffer III (BD Biosciences) on ice, washed four times in FACS buffer, and stained. Samples were acquired on a BD Fortessa instrument (BD Biosciences) and analyzed using FlowJo v10 (TreeStar). Samples were gated on single cells using FSC-A/FSC-H and SSC-A/SSC-H and, to exclude debris, on FSC-A/SSC-A. Dead cells were excluded before gating based on antibody staining to identify cell populations as described in the respective figures. Intestinal eosinophils were generally identified as CD45^+^CD11b^+^MHC-II^–^Siglec-F^+^SSC^hi^ cells.

### Cell sorting

Single-cell suspensions were stained as described above. Dead cells and doublets were excluded. For sorting multiple intestinal populations from the small intestine (Fig. S2 D), unselected cells were used to sort total CD45^+^ cells, epithelial cells (CD45^–^EPCAM^+^), fibroblasts (CD45^–^EPCAM^–^CD31^–^CD29^+^) and endothelial cells (CD45^–^EPCAM^–^CD31^+^). Magnetic bead selection with anti-CD11b and anti-CD11c (Miltenyi Biotech) was performed according to protocol over LS columns to separate myeloid (CD11b^+^ and/or CD11c^+^) from lymphoid cells. Myeloid cells were sorted into eosinophils (CD45^+^SSC^hi^MHCII^–^SiglecF^+^), monocytes (CD45^+^SSC^lo^MHCII^–^CD11b^+^Ly6C^+^), macrophages (CD45^+^CD11b^+^MHCII^+^CD64^+^F480^+^), and dendritic cells (CD45^+^CD64^–^F480^–^MHCII^+^CD11c^+^CD103^+^). Lymphoid cells were sorted into B cells (CD45^+^CD3^–^B220^+^), αβ T cells (CD45^+^CD3^+^B220^+^CD90^+^TCRb^+^), γδ T cells (CD45^+^CD3^+^B220^+^CD90^+^TCRgd^+^), and other lymphoid cells (CD45^+^CD3^–^B220^–^CD90^+^). Eosinophils from the small intestine and bone marrow that were sorted for RNA sequencing were additionally stained with DAPI (Sigma-Aldrich) to exclude any cells that died between staining and sorting. For eosinophil-only sorting, cells were preselected with anti-CD11b microbeads for all tissues except bone marrow, which was used unselected. Cell sorting was conducted on Aria III or Fusion instruments (BD Bioscience) through a 100-µm nozzle using high-purity settings.

### In vitro culture of primary mouse eosinophils

Eosinophils were sorted into FCS, centrifuged, and resuspended at 1 × 10^6^ cells/ml in RPMI medium with 10% FCS, penicillin/streptomycin, and 2 ng/ml IL-5. 50,000 cells per sample were seeded, and eosinophils were treated as described in the figure legends with FICZ (#SML1489; Sigma-Aldrich), DMSO (Sigma-Aldrich), or CH223191 (#S7711; Sigma-Aldrich) and cultured for indicated times at 37°C, 5% CO_2_.

### Eosinophil adhesion assay

Plates were coated with 20 μl/96-well of rat collagen 1 (#3447-020-01; R&D Systems), Matrigel (Cultrex RGF BME Type II; #3533-005-02; R&D Systems), 0.1 mg/ml laminin (#11243217001; Roche), or 1 mg/ml fibronectin (#F4759; Sigma-Aldrich) on ice and then incubated at 37°C for 30 min. Culture medium (IMDM, 10% FCS, and penicillin/streptomycin) was added, and plates were stored in the cell culture incubator overnight. FACS-sorted small intestinal eosinophils were seeded onto coated wells at 50,000 cells per 100 μl per well in medium supplemented with 50 pM FICZ and 10 ng/ml IL-5. Cells were incubated for 16 h, and nonadherent cells were washed away three times with 200 μl HBSS. Adherent cells were quantified using CyQUANT (#C35007; Invitrogen) according to the manufacturer’s instructions and normalized to a cell standard curve and the number of seeded cells.

### Gelatin degradation assay

Gelatin degradation was measured using the QCM Gelatin Invadopodia Assay (#ECM670; Sigma-Aldrich) according to the manufacturer’s instructions. 8-well chamber slides were coated and stored with culture medium in the incubator overnight. Sorted small intestinal eosinophils were seeded at 30,000 cells per well in 400 μl medium supplemented with 50 pM FICZ and 10 ng/ml IL-5. Cells were incubated for 20 h, washed, fixed, and stained with TRITC-phalloidin. 8–10 images per well were acquired on a Zeiss Invert710 confocal microscope with a 10× objective. Image analysis was conducted in Fiji. The total area of gelatin degradation was divided by the number of cells on each image, and results were averaged across 8–10 images per sample.

### RNA isolation

Cell pellets were resuspended in Trizol (#15596026; Invitrogen), and RNA was isolated according to protocol. For BMDEo culture time points and stimulated BMDEo, RNA was isolated from ∼200,000 cells/sample. For BMDEo gene expression analysis in Figs. 4 I and S3 G, RNA was isolated from 2 × 10^6^ cells/sample. For RNA sequencing, RNA was isolated with TRI Reagent and the RiboPure RNA Purification Kit (#AM1924; Thermo Fisher Scientific) immediately after sorting. RNA quality and quantity were determined using the Agilent 2100 Bioanalyzer with the Agilent RNA 6000 Pico Kit. Only samples with RNA integrity number ≥7 were used for RNA sequencing.

### qPCR

RNA was reverse transcribed using the High-Capacity cDNA Reverse Transcription Kit (Thermo Fisher Scientific). The cDNA served as a template for the amplification of genes of interest and housekeeping genes by real-time qPCR, using TaqMan Gene Expression Assays (Applied Biosystems), universal PCR Master Mix (Applied Biosystems), and the QuantStudio 7 System (Applied Biosystems). For gene expression analysis in BMDEo (Figs. 4 I and S3 G), real-time qPCR was conducted using Power SYBR Green PCR Master Mix (Thermo Fisher Scientific). mRNA expression was determined using the ΔC_T_ method, normalizing to hypoxanthine-guanine phosphoribosyltransferase (*Hprt*) gene expression.

### RNA sequencing and data processing

RNA samples were converted into cDNA using the NuGEN Ovation RNA-Seq System v2. Illumina-compatible libraries were produced using the NuGEN Ovation Ultralow Library System v2. Sequencing was performed on the Illumina HiSeq 4000 with single-ended reads of ≥75 bp. Fastq files were aligned to the GRCm38 Ensembl Release 86 mouse genome using the nextflow package nf-core/rnaseq to generate gene counts. Differential gene discovery was done using the Bioconductor package DESeq2 (Love et al., 2014). Comparisons between WT and *Ahr*^–/–^ eosinophils were adjusted for sex. We recorded as differentially expressed those genes with adjusted P values <0.05. We also relied on DESeq2 for principal component analysis of variance stabilized expression values. All computations were performed in R v4.0.3 (2020-10-10). Datasets have been deposited to GEO under accession numbers GSE185070 (WT eosinophils from bone marrow and small intestine) and GSE173831 (small intestinal eosinophils from WT and *Ahr*^–/–^).

### Comparison of datasets

To compare datasets, variance stabilized counts from dataset 1 (bone marrow versus small intestinal eosinophils) were normalized to dataset 2 (WT versus *Ahr*^–/–^, both from small intestine) by normalizing the average of small intestinal eosinophils from dataset 1 to that of WT female eosinophils from dataset 2 (biologically equivalent samples) for each gene. For hierarchical clustering, only genes with differential expression in dataset 2 (WT versus *Ahr*^–/–^ eosinophils; *n* = 1,292 genes) and for which counts data were also available in dataset 1 (removing three genes) were included.

### Hierarchical clustering

Hierarchical clustering was conducted using Morpheus (https://software.broadinstitute.org/morpheus) by clustering differentially expressed genes with an average linkage method and one minus Pearson correlation metric. For display, gene expression values were transformed using a relative color scheme showing row minimum to row maximum (the minimum and maximum expression values of each gene).

### GSEA

We used all genes ordered by log_2_(FC) as the ranking for GSEA conducted in GSEA v4.1.0 (Subramanian et al., 2005). To determine enrichment of canonical pathways (C2.all.v7.4), mouse gene symbols were remapped to human orthologs. Leading-edge analysis was used to determine and exclude overlapping gene sets. Selected gene sets with P < 0.05 and false discovery rate < 0.25 are shown.

### Gene ontology analysis

Differentially expressed genes (adjusted P value <0.05) from different comparisons were analyzed using DAVID v6.8 (Huang da et al., 2009) to determine Kegg and gene ontology pathways and Uniprot keywords. Selected pathways are shown. DAVID was also used to filter transcription factors (gene ontology term 0003700) from the lists of differentially expressed genes.

### Statistical analysis and graphing

Details of the statistical tests applied and number of replicates are described in the corresponding figure legends. All data points and *n* values reflect biological replicates (mice or human subjects). Statistical analysis was performed using Prism 9 (GraphPad) software.

### Online supplemental material

Fig. S1 shows the gating and cell sorting strategy for eosinophils from the bone marrow and small intestine, a heatmap of differentially expressed genes, and examples of genes for several enriched pathways. Genes with the highest absolute expression and ≥10-fold change between bone marrow and small intestine are shown. Fig. S2 depicts the AHR-TdTomato mouse construct and eosinophil gating strategy from different tissues. It also shows AHR expression by AHR-TdTomato fluorescence and qPCR across different intestinal cell populations. Fig. S3 compares the gene expression between WT and *Ahr*^–/–^ small intestinal eosinophils. It shows genes with differential expression between male and female WT samples, a heatmap of differentially expressed genes between WT and *Ahr*^*−/−*^ eosinophils, selected enriched pathways, gene ontology analysis, comparative qPCR data from BMDEo, and differentially expressed transcription factors. Fig. S4 shows validation of eosinophil-specific AHR deletion in Epx^Cre/+^*Ahr*^fl/fl^ mice as well as eosinophil frequencies across different tissues and EoP frequencies in the BM. It also depicts Ki67 staining of intestinal eosinophils and representative flow cytometry plots of EdU staining in intestinal eosinophils and of adoptively transferred eosinophils retrieved from the small intestine. Fig. S5 shows CD80 and CD274 staining of intestinal eosinophils as well as staining for T cells and monocyte-macrophage populations. It also shows worm burden, immune cell staining, and representative histological images after *H.p.* infection.
